# Supplementary material for: Signatures of hierarchical temporal processing in the mouse visual system
Source: PLoS Comput Biol. 2024 Aug 22;20(8):e1012355. doi: 10.1371/journal.pcbi.1012355 (PMC11373856; doi:10.1371/journal.pcbi.1012355)
Supplement: S1 Fig — (A) Sorted units available for analysis after spike sorting process (“valid wave-forms”), after applying filters of the AllenSDK (“quality metrics”), after selecting only units of the “Functional Connectivity set” and after ensuring that the recordings of the selected “stimuli” for each unit are long enough and do not include invalid spike times (Materials and methods). (B) Numbers of units for each session and for each area available for analysis after filtering. (PDF) [file pcbi.1012355.s001.pdf]

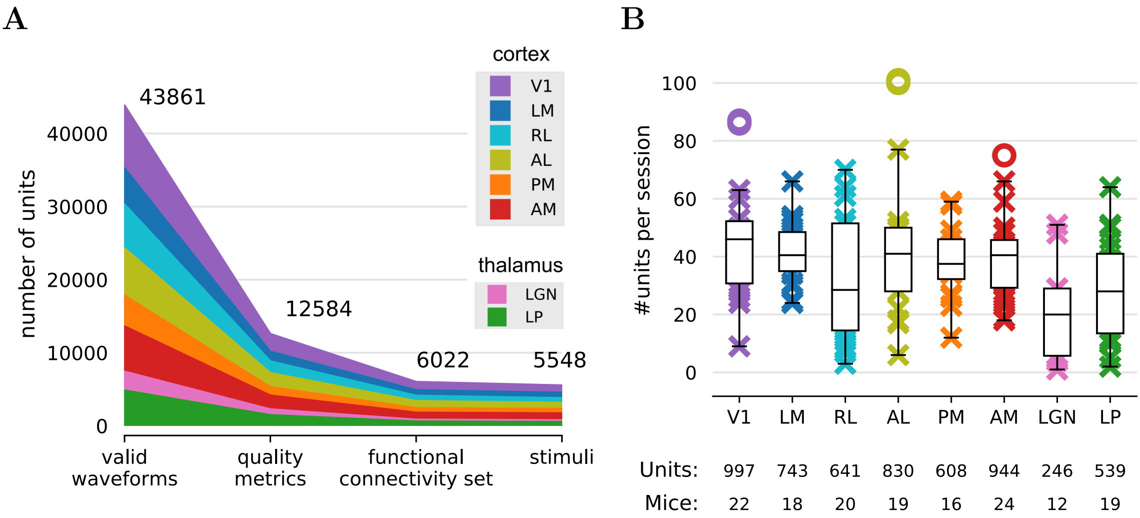

Figure S1. Number of available units for the analysis for the *Functional Connectivity* data set. (A) Sorted units available for analysis after spike sorting process ("valid wave-forms"), after applying filters of the AllenSDK ("quality metrics"), after selecting only units of the "Functional Connectivity set" and after ensuring that the recordings of the selected "stimuli" for each unit are long enough and do not include invalid spike times (Methods). **(B)** Numbers of units for each session and for each area available for analysis after filtering.
