# Supplementary material for: Signatures of hierarchical temporal processing in the mouse visual system
Source: PLoS Comput Biol. 2024 Aug 22;20(8):e1012355. doi: 10.1371/journal.pcbi.1012355 (PMC11373856; doi:10.1371/journal.pcbi.1012355)
Supplement: S2 Fig — Histograms of the correlation timescale τC, the information timescale τR and the predictability Rtot (diagonal), as well as scatter plots of one measure against the other (y-axes refer to scatter plots, no axes shown for histograms) for all analyzed units. Scatter plots are overlaid with kernel density estimations, where lines indicate regions of equal probability. Correlation and information timescales are shown in log scale. Timescales are positively correlated (Pearson correlation), whereas predictability is weakly negatively correlated with the timescales. (PDF) [file pcbi.1012355.s002.pdf]

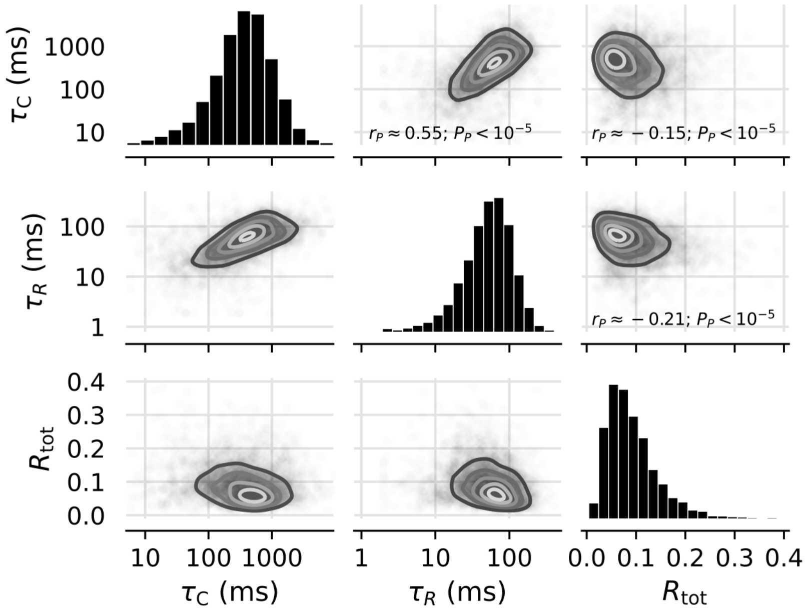

Figure S2. Relation between correlation and information timescales, as well as predictability across all sorted units. Histograms of the correlation timescale τ_C, the information timescale τ_R and the predictability R_{tot} (diagonal), as well as scatter plots of one measure against the other (y-axes refer to scatter plots, no axes shown for histograms) for all analyzed units. Scatter plots are overlaid with kernel density estimations, where lines indicate regions of equal probability. Correlation and information timescales are shown in log scale. Timescales are positively correlated (Pearson correlation), whereas predictability is weakly negatively correlated with the timescales.
