# Supplementary material for: Signatures of hierarchical temporal processing in the mouse visual system
Source: PLoS Comput Biol. 2024 Aug 22;20(8):e1012355. doi: 10.1371/journal.pcbi.1012355 (PMC11373856; doi:10.1371/journal.pcbi.1012355)
Supplement: S3 Fig — Scatter plots of a measure of timescale or predictability versus common firing statistics such as the average firing rate, median inter-spike-interval (ISI) and coefficient of variation (CV) for all analyzed units. Scatter plots are overlaid with kernel density estimations, where lines indicate regions of equal probability. Correlation and information timescales, as well as median ISIs are shown in log scale. The firing rate is mainly positively correlated with the correlation timescale, and negatively correlated with the predictability (Pearson correlation). The median ISI is mainly correlated with the information timescale, whereas the CV is strongly correlated with the predictability. (PDF) [file pcbi.1012355.s003.pdf]

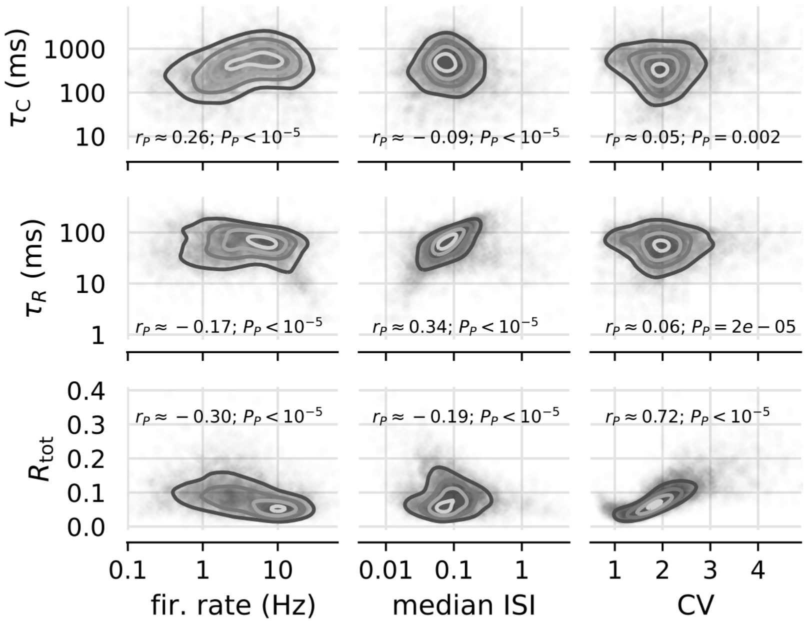

Figure S3. Relation of correlation and information timescales, as well as predictability to common firing statistics. Scatter plots of a measure of timescale or predictability versus common firing statistics such as the average firing rate, median inter-spike-interval (ISI) and coefficient of variation (CV) for all analyzed units. Scatter plots are overlaid with kernel density estimations, where lines indicate regions of equal probability. Correlation and information timescales, as well as median ISIs are shown in log scale. The firing rate is mainly positively correlated with the correlation timescale, and negatively correlated with the predictability (Pearson correlation). The median ISI is mainly correlated with the information timescale, whereas the CV is strongly correlated with the predictability.
