# Supplementary material for: Signatures of hierarchical temporal processing in the mouse visual system
Source: PLoS Comput Biol. 2024 Aug 22;20(8):e1012355. doi: 10.1371/journal.pcbi.1012355 (PMC11373856; doi:10.1371/journal.pcbi.1012355)
Supplement: S4 Fig — Firing rate and median interspike-interval (ISI) of sorted units tend to increase with hierarchy score, but have lower Pearson correlation coefficients rP (with higher p-values PP), as well as Spearman correlation coefficients rS, compared to timescales or predictability on the same data set in Fig 2. The coefficient of variation (CV), in contrast, is not correlated with the hierarchy score. (PDF) [file pcbi.1012355.s004.pdf]

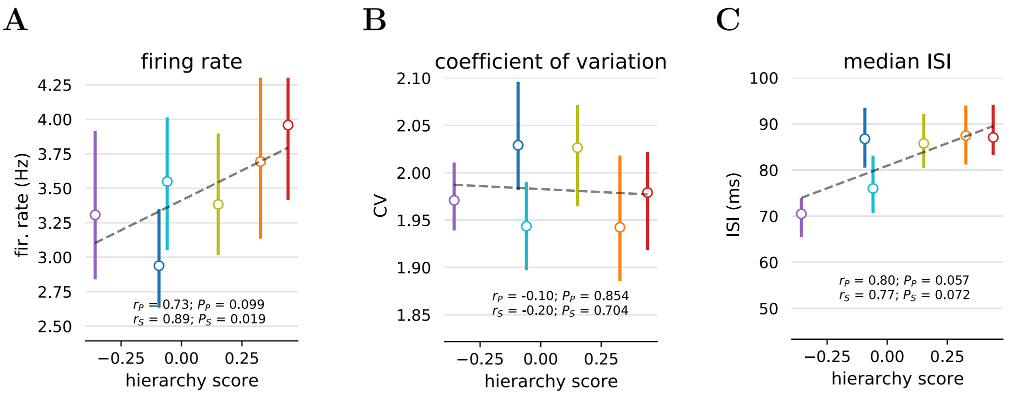

Figure S4. Correlation between single neuron firing statistics and hierarchy score for natural movie stimulation in the *Functional Connectivity* data set. Firing rate and median interspike-interval (ISI) of sorted units tend to increase with hierarchy score, but have lower Pearson correlation coefficients r_P (with higher p-values P_P), as well as Spearman correlation coefficients r_S, compared to timescales or predictability on the same data set in Fig. 2. The coefficient of variation (CV), in contrast, is not correlated with the hierarchy score.
