# Supplementary material for: Signatures of hierarchical temporal processing in the mouse visual system
Source: PLoS Comput Biol. 2024 Aug 22;20(8):e1012355. doi: 10.1371/journal.pcbi.1012355 (PMC11373856; doi:10.1371/journal.pcbi.1012355)
Supplement: S5 Fig — (A) For a single timescale fit, the median estimated timescale (over all cortical units) increases monotonously with maximum lag Tmax, independent of the minimum time lag Tmin used for fitting (light and dark blue lines). In contrast, when using a two-timescale fit, the median estimated correlation timescale remains consistent for sufficiently large max lags Tmax > 1000 ms, for both, Tmin = 5 ms and Tmin = 30 ms (light and dark green lines). However, timescales are larger for Tmin = 5 ms, because often fits are flattened by negative autocorrelation for short time lags, which are mostly excluded with Tmin = 30 ms. (B) The Pearson correlation coefficient rP between an area’s median correlation timescale and anatomical hierarchy score (c.f. Fig 2D) is consistently high if Tmax is chosen sufficiently high. (C) Similarly, for sufficiently large Tmax, the p-value of the fit is consistently below 0.05. For all plots, red dot indicates the Tmax = 10s used for the main analyses. Timescales were estimated for the natural movie condition of the Functional Connectivity data set. (PDF) [file pcbi.1012355.s005.pdf]

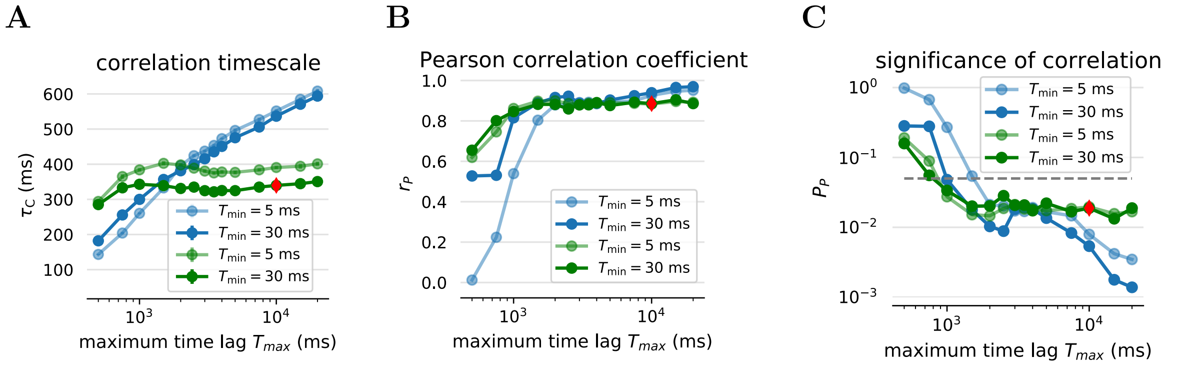

Figure S5. For single timescale fit, median correlation timescale and hierarchy score correlation are sensitive to the maximum time lag used for fitting. (A) For a single timescale fit, the median estimated timescale (over all cortical units) increases monotonously with maximum lag $T_{\max}$, independent of the minimum time lag $T_{\min}$ used for fitting (light and dark blue lines). In contrast, when using a two-timescale fit, the median estimated correlation timescale remains consistent for sufficiently large max lags $T_{\max} > 1000\,\text{ms}$, for both, $T_{\min} = 5\,\text{ms}$ and $T_{\min} = 30\,\text{ms}$ (light and dark green lines). However, timescales are larger for $T_{\min} = 5\,\text{ms}$, because often fits are flattened by negative autocorrelation for short time lags, which are mostly excluded with $T_{\min} = 30\,\text{ms}$. **(B)** The Pearson correlation coefficient r_P between an area's median correlation timescale and anatomical hierarchy score (c.f. Fig. 2D) is consistently high if $T_{\max}$ is chosen sufficiently high. **(C)** Similarly, for sufficiently large $T_{\max}$, the p-value of the fit is consistently below 0.05. For all plots, red dot indicates the $T_{\max} = 10\,\text{s}$ used for the main analyses. Timescales were estimated for the natural movie condition of the *Functional Connectivity* data set.
