# Supplementary material for: Signatures of hierarchical temporal processing in the mouse visual system
Source: PLoS Comput Biol. 2024 Aug 22;20(8):e1012355. doi: 10.1371/journal.pcbi.1012355 (PMC11373856; doi:10.1371/journal.pcbi.1012355)
Supplement: S6 Fig — To assess how estimated correlation timescales differ for the single and two-timescale fits, we here show the distribution of fitted timescales for the single timescale fit (blue), and the two fitted timescales for the two-timescale fit (orange). For the latter, we show both, the selected timescale (see Materials and methods), which yields the estimate of the correlation timescale, and the rejected timescale. The single timescale is generally larger than the selected timescale of the two-timescale fit, because it also accounts for a potential slow decay of autocorrelation on long timescales. For the two-timescale fit, in contrast, it is mostly the rejected timescale that accounts for the long timescales, since the rejected timescales are generally much larger. (PDF) [file pcbi.1012355.s006.pdf]

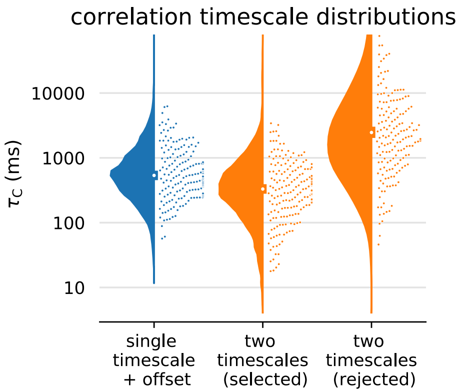

Figure S6. Distribution of fitted timescales for the single and two-timescales fit. To assess how estimated correlation timescales differ for the single and two-timescale fits, we here show the distribution of fitted timescales for the single timescale fit (blue), and the two fitted timescales for the two-timescale fit (orange). For the latter, we show both, the selected timescale (see Methods), which yields the estimate of the correlation timescale, and the rejected timescale. The single timescale is generally larger than the selected timescale of the two-timescale fit, because it also accounts for a potential slow decay of autocorrelation on long timescales. For the two-timescale fit, in contrast, it is mostly the rejected timescale that accounts for the long timescales, since the rejected timescales are generally much larger.
