# Supplementary material for: Signatures of hierarchical temporal processing in the mouse visual system
Source: PLoS Comput Biol. 2024 Aug 22;20(8):e1012355. doi: 10.1371/journal.pcbi.1012355 (PMC11373856; doi:10.1371/journal.pcbi.1012355)
Supplement: S7 Fig — To demonstrate the effect of the fitting range on the inferred hierarchy of correlation timescales τC, we repeated the same analysis from Fig 2D for a smaller maximum time lag Tmax = 500 ms, and different minimum time lags Tmin. For this choice of Tmax, no hierarchy is found, and median values of τC are in general much lower (dots, bars indicate bootstrapping confidence intervals on median). This is found for all choices of Tmin, indicating that this is primarily caused by omitting larger time lags T > Tmax during fitting. Here, correlation timescales were computed for spiking activity under natural movie stimulation in the Functional Connectivity data set. Moreover, τC was obtained from a single timescale fit to enable a comparison to previous analyses (c.f. Extended Data Fig 9 in [32]), but we obtained a similar result also for the two-timescale fit, although this approach is generally much more robust to the choice of fitting range (c.f. S5 Fig). (PDF) [file pcbi.1012355.s007.pdf]

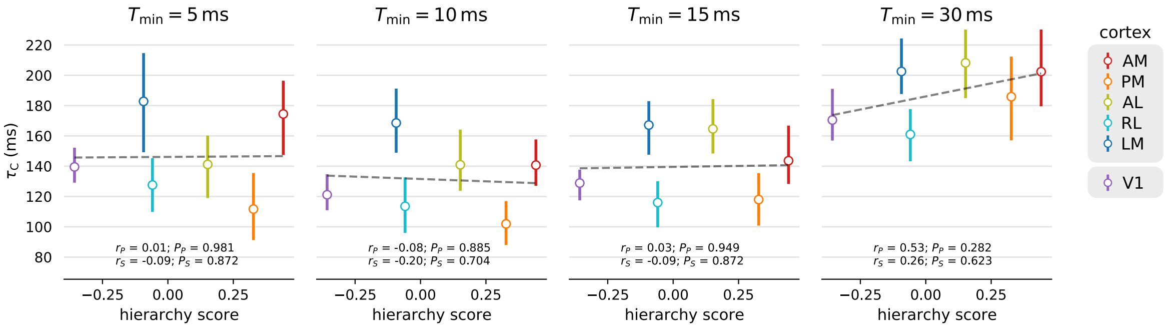

Figure S7. No hierarchy of correlation timescales is found for a small fitting range. To demonstrate the effect of the fitting range on the inferred hierarchy of correlation timescales τ_C, we repeated the same analysis from Fig. 2D for a smaller maximum time lag $T_{\mathrm{max}} = 500\,\mathrm{ms}$, and different minimum time lags T_{min}. For this choice of T_{max}, no hierarchy is found, and median values of τ_C are in general much lower (dots, bars indicate bootstrapping confidence intervals on median). This is found for all choices of T_{min}, indicating that this is primarily caused by omitting larger time lags $T > T_{\mathrm{max}}$ during fitting. Here, correlation timescales were computed for spiking activity under natural movie stimulation in the *Functional Connectivity* data set. Moreover, τ_C was obtained from a single timescale fit to enable a comparison to previous analyses (c.f. Extended Data Fig. 9 in [32]), but we obtained a similar result also for the two-timescale fit, although this approach is generally much more robust to the choice of fitting range (c.f. Supplementary Fig. S5).
