# Supplementary material for: Signatures of hierarchical temporal processing in the mouse visual system
Source: PLoS Comput Biol. 2024 Aug 22;20(8):e1012355. doi: 10.1371/journal.pcbi.1012355 (PMC11373856; doi:10.1371/journal.pcbi.1012355)
Supplement: S8 Fig — For a large fitting range with Tmax = 10 s, we consistently find a hierarchy of correlation timescales τC, whereas Tmin only slightly affects the exact layout of the hierarchy, and the median values of τC (c.f. S5 Fig). Here, correlation timescales were computed for spiking activity under natural movie stimulation in the Functional Connectivity data set. Moreover, timescales where obtained using the two-timescale fitting procedure (Materials and methods), because this is the analysis used for all main results obtained in this paper. (PDF) [file pcbi.1012355.s008.pdf]

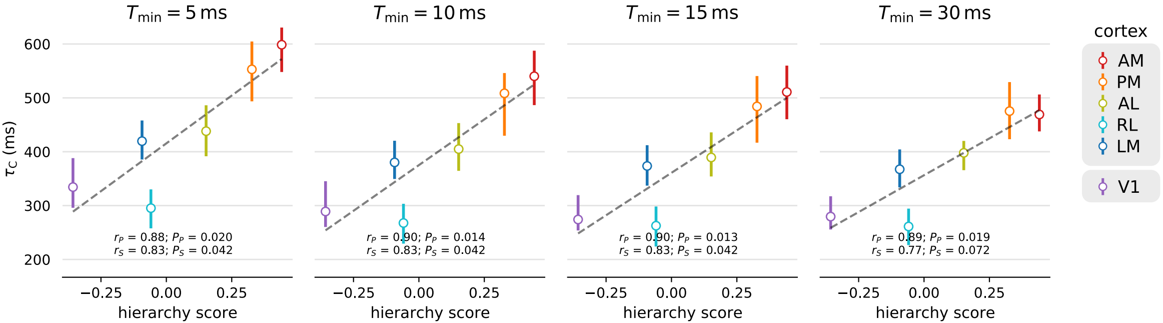

Figure S8. For a sufficiently large fitting range, the hierarchy of timescales is found independent of the exclusion of small time lags during fitting. For a large fitting range with $T_{\max} = 10\,\mathrm{s}$, we consistently find a hierarchy of correlation timescales τ_C, whereas $T_{\min}$ only slightly affects the exact layout of the hierarchy, and the median values of τ_C (c.f. Supplementary Fig. S5). Here, correlation timescales were computed for spiking activity under natural movie stimulation in the *Functional Connectivity* data set. Moreover, timescales where obtained using the two-timescale fitting procedure (Methods), because this is the analysis used for all main results obtained in this paper.
