# Supplementary material for: Signatures of hierarchical temporal processing in the mouse visual system
Source: PLoS Comput Biol. 2024 Aug 22;20(8):e1012355. doi: 10.1371/journal.pcbi.1012355 (PMC11373856; doi:10.1371/journal.pcbi.1012355)
Supplement: S9 Fig — Measured autocorrelation functions (grey line) for units in the Functional Connectivity data set under natural movie stimulation. Black dots and green lines indicate single and two-timescale fits, respectively, with the inferred timescale stated in the corresponding color. (PDF) [file pcbi.1012355.s009.pdf]

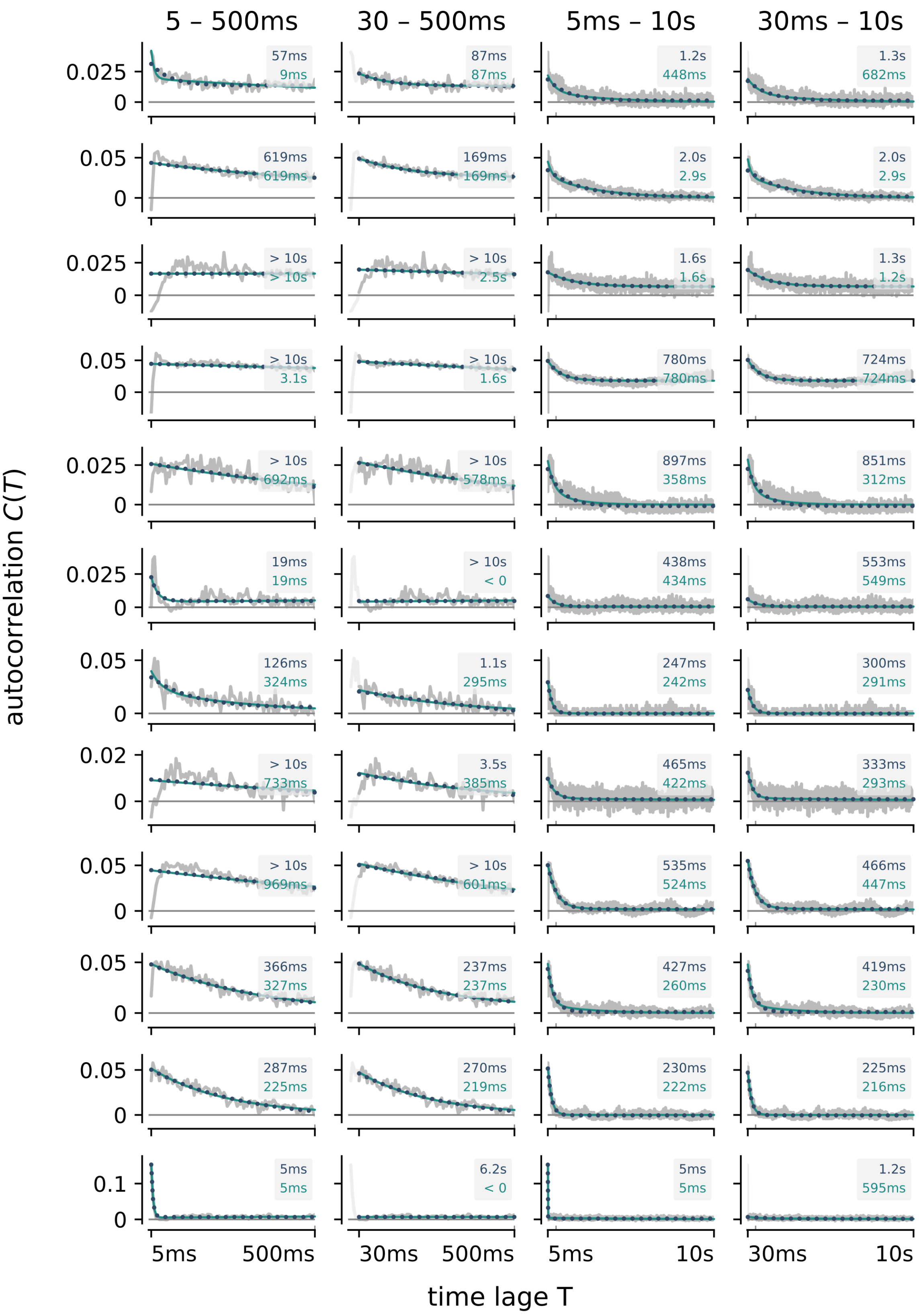

Figure S9. Randomly selected examples of autocorrelation functions and single and two-timescale fits for different fitting ranges. Measured autocorrelation functions (grey line) for units in the *Functional Connectivity* data set under natural movie stimulation. Black dots and green lines indicate single and two-timescale fits, respectively, with the inferred timescale stated in the corresponding color.
