# Supplementary material for: Signatures of hierarchical temporal processing in the mouse visual system
Source: PLoS Comput Biol. 2024 Aug 22;20(8):e1012355. doi: 10.1371/journal.pcbi.1012355 (PMC11373856; doi:10.1371/journal.pcbi.1012355)
Supplement: S10 Fig — Similar to the correlation timescale, we excluded past ranges smaller than some Tmin when computing the information timescale τR to exclude short-term effects like refractoriness and tonic firing (Materials and methods). Here, we show median information timescales for cortical areas, each time for a different choice of minimal past range Tmin. Notably, differences between higher cortical areas (LM, AL, PM, AM) are only visible when excluding past ranges smaller than Tmin = 30 ms. Information timescales were computed for spiking activity under natural movie stimulation in the Functional Connectivity data set. (PDF) [file pcbi.1012355.s010.pdf]

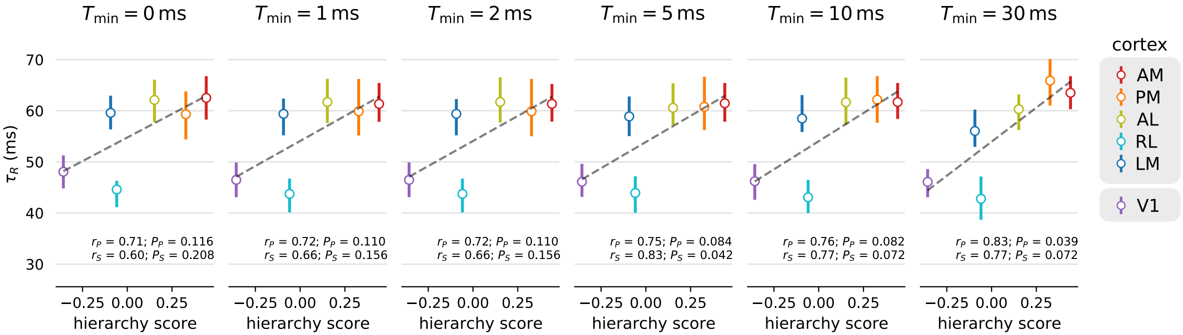

Figure S10. Hierarchy of information timescale relies on excluding small past ranges from the analysis. Similar to the correlation timescale, we excluded past ranges smaller than some $T_{\min}$ when computing the information timescale τ_R to exclude short-term effects like refractoriness and tonic firing (Methods). Here, we show median information timescales for cortical areas, each time for a different choice of minimal past range $T_{\min}$. Notably, differences between higher cortical areas (LM, AL, PM, AM) are only visible when excluding past ranges smaller than $T_{\min} = 30\,\text{ms}$. Information timescales were computed for spiking activity under natural movie stimulation in the *Functional Connectivity* data set.
