# Supplementary material for: Signatures of hierarchical temporal processing in the mouse visual system
Source: PLoS Comput Biol. 2024 Aug 22;20(8):e1012355. doi: 10.1371/journal.pcbi.1012355 (PMC11373856; doi:10.1371/journal.pcbi.1012355)
Supplement: S11 Fig — (A–C) As for recorded activity in the Functional Connectivity data set, the medians of all measures differ significantly for different structural groups (thalamus, primary visual cortex and higher cortical), with the same ordering as before. Black boxes indicate the median over sorted units of the different structural groups, whereas coloured dots indicate the median for individual areas. Bars indicate standard deviation on the median obtained from bootstrapping. (D–F) Measures across the cortical hierarchy show the same general increase as for the Functional Connectivity data set. However, median correlation and information timescales show higher variability, and thus we find smaller correlation coefficients (dashed line, Pearson and Spearman correlation coefficients and corresponding p-values shown below). (PDF) [file pcbi.1012355.s011.pdf]

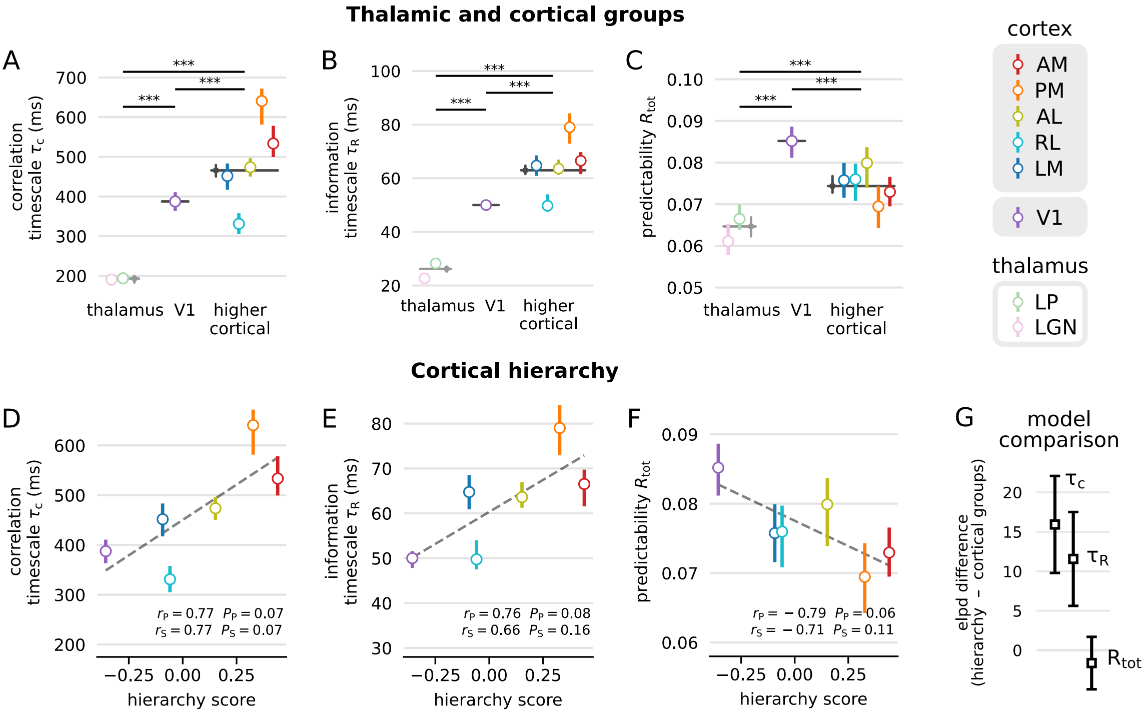

Figure S11. Timescale and predictability of different brain areas under natural movie stimulation in the *Brain Observatory 1.1* data set. (A–C) As for recorded activity in the *Functional Connectivity* data set, the medians of all measures differ significantly for different structural groups (thalamus, primary visual cortex and higher cortical), with the same ordering as before. Black boxes indicate the median over sorted units of the different structural groups, whereas coloured dots indicate the median for individual areas. Bars indicate standard deviation on the median obtained from bootstrapping. **(D–F)** Measures across the cortical hierarchy show the same general increase as for the *Functional Connectivity* data set. However, median correlation and information timescales show higher variability, and thus we find smaller correlation coefficients (dashed line, Pearson and Spearman correlation coefficients and corresponding p-values shown below).
