# Supplementary material for: Signatures of hierarchical temporal processing in the mouse visual system
Source: PLoS Comput Biol. 2024 Aug 22;20(8):e1012355. doi: 10.1371/journal.pcbi.1012355 (PMC11373856; doi:10.1371/journal.pcbi.1012355)
Supplement: S12 Fig — (A–C) As for recorded activity under natural stimulation, the medians of correlation and information timescales differ significantly between different structural groups (thalamus, primary visual cortex and higher cortical), with the same ordering as before. However, the difference in median predictability between V1 and higher cortical areas is not significant for spontaneous activity. Black boxes indicate the median over sorted units of the different structural groups, whereas coloured dots indicate the median for individual areas. Bars indicate standard deviation on the median obtained from bootstrapping. (D–F) Measures across the cortical hierarchy show the same general trend as for natural movie stimulation. However, in general, measures for different areas are more similar, leading to smaller correlation values with the hierarchy score and higher p-values (dashed line, Pearson and Spearman correlation coefficients and corresponding p-values shown below). (PDF) [file pcbi.1012355.s012.pdf]

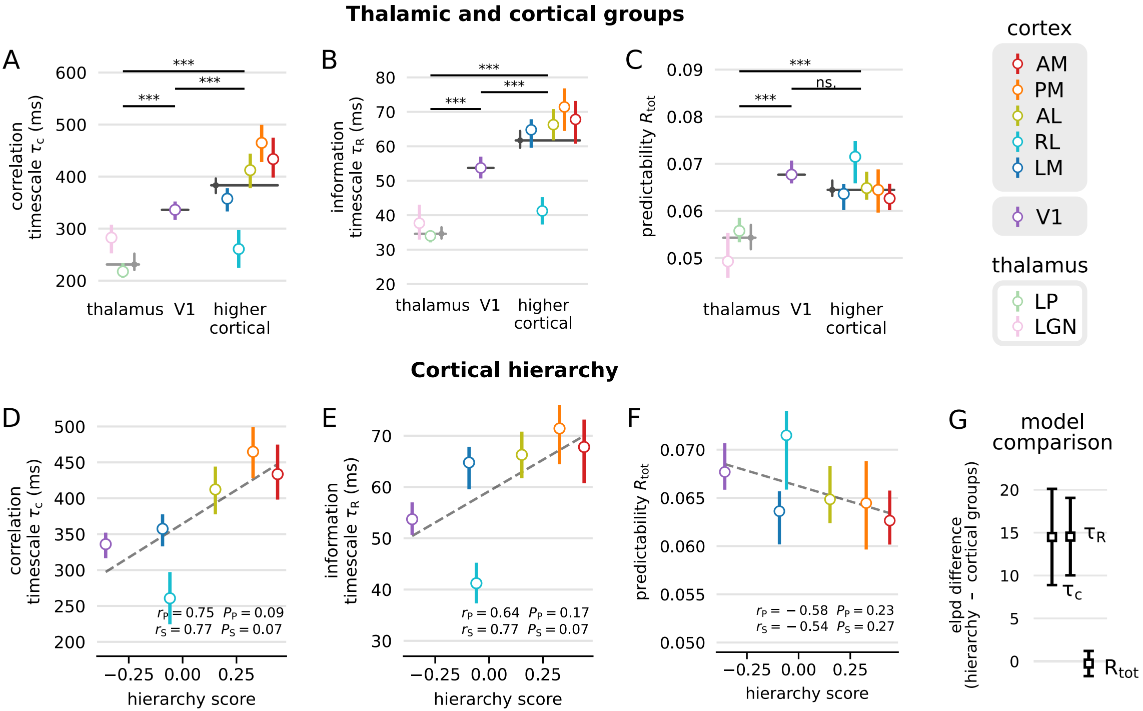

Figure S12. Timescales and predictability of different brain areas for *spontaneous activity* in the *Functional Connectivity* data set. (A–C) As for recorded activity under natural stimulation, the medians of correlation and information timescales differ significantly between different structural groups (thalamus, primary visual cortex and higher cortical), with the same ordering as before. However, the difference in median predictability between V1 and higher cortical areas is not significant for spontaneous activity. Black boxes indicate the median over sorted units of the different structural groups, whereas coloured dots indicate the median for individual areas. Bars indicate standard deviation on the median obtained from bootstrapping. **(D–F)** Measures across the cortical hierarchy show the same general trend as for natural movie stimulation. However, in general, measures for different areas are more similar, leading to smaller correlation values with the hierarchy score and higher p-values (dashed line, Pearson and Spearman correlation coefficients and corresponding p-values shown below).
