# Supplementary material for: Signatures of hierarchical temporal processing in the mouse visual system
Source: PLoS Comput Biol. 2024 Aug 22;20(8):e1012355. doi: 10.1371/journal.pcbi.1012355 (PMC11373856; doi:10.1371/journal.pcbi.1012355)
Supplement: S13 Fig — To assess whether timescales and predictability relate to the anatomical cortical hierarchy, a linear relationship between an area’s median and anatomical hierarchy score was modelled with slope θhs. (A) (Top) For the correlation timescale, the 95% posterior credible interval of the mean hierarchy score slope μθhs across all mice is positive (black bar, red dot indicates median), indicating that there is an increase in median correlation timescales with the hierarchy score. (Bottom) On the level of individual mice, posteriors indicate the same effect, but are more diverse (colors indicate different mice). In particular, for some mice the posteriors also attribute probability to zero or negative slopes, which could be either due to increased uncertainty due to the smaller sampling size, or an incomplete sampling of the areas for individual mice. (B) For the information timescale τR, the posterior credible interval of the mean slope is also positive. (C) For predictability, in contrast, the credible interval is negative, indicating a credible decrease in predictability with hierarchy score. (D–F) Very similar results are obtained for the Brain Observatory data set. (G) For spontaneous activity and the correlation timescale, the posterior of the mean slope is very similar to the natural movie conditions, but variability across mice is larger. (H) For the information timescale, in contrast, the posterior indicates a smaller slope. (I) For predictability, the credible interval even contains a zero slope, indicating that predictability does not necessarily decrease along the anatomical hierarchy for spontaneous activity. (PDF) [file pcbi.1012355.s013.pdf]

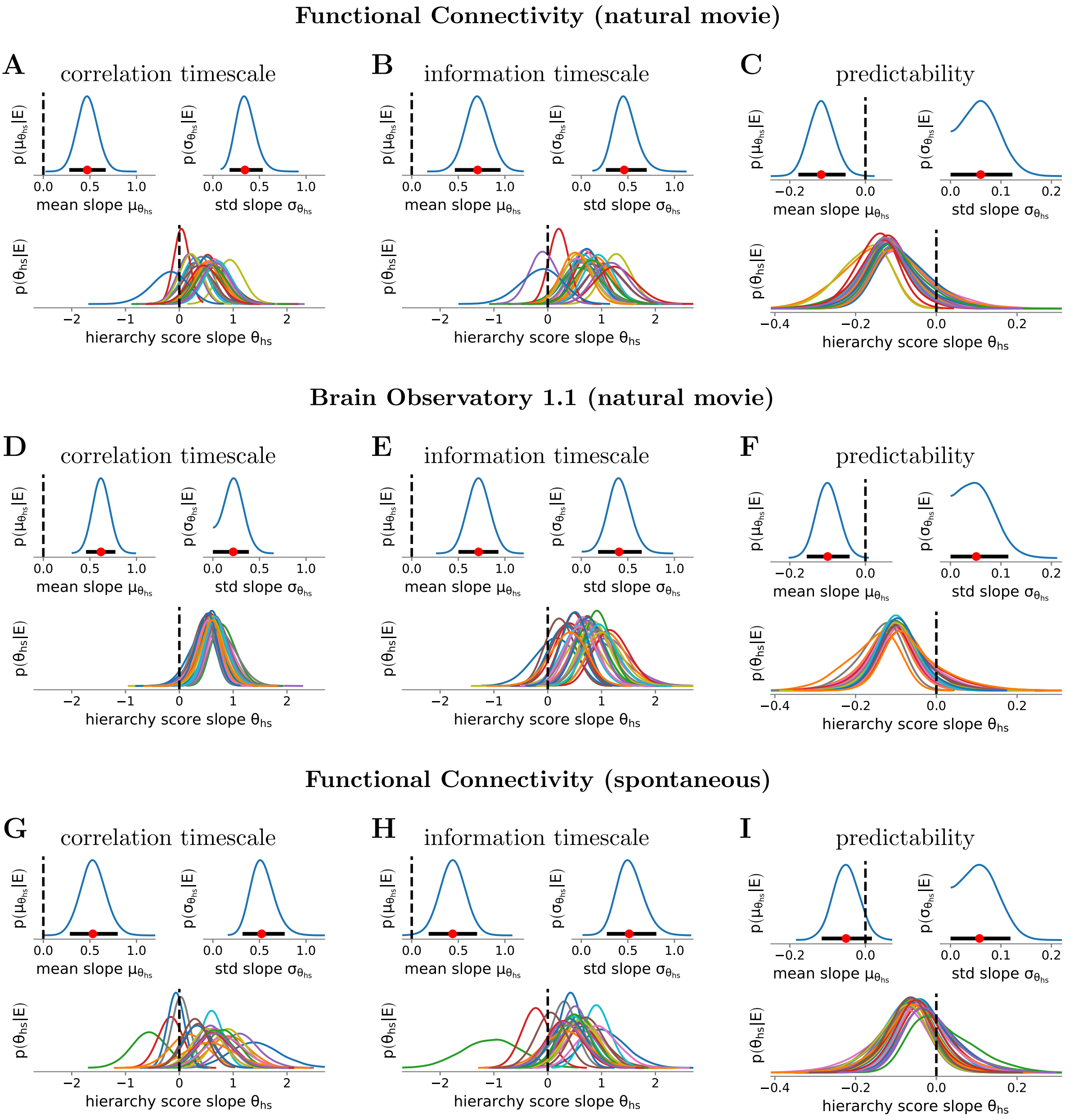

Figure S13. Posterior distributions of the hierarchy score slope reveal a significant increase in timescales and decrease in predictability with hierarchy score. To assess whether timescales and predictability relate to the anatomical cortical hierarchy, a linear relationship between an area's median and anatomical hierarchy score was modelled with slope θ_{hs}. **(A)** (Top) For the correlation timescale, the 95% posterior credible interval of the mean hierarchy score slope $\mu_{\theta_{\mathrm{hs}}}$ across all mice is positive (black bar, red dot indicates median), indicating that there is an increase in median correlation timescales with the hierarchy score. (Bottom) On the level of individual mice, posteriors indicate the same effect, but are more diverse (colors indicate different mice). In particular, for some mice the posteriors also attribute probability to zero or negative slopes, which could be either due to increased uncertainty due to the smaller sampling size, or an incomplete sampling of the areas for individual mice. **(B)** For the information timescale τ_R, the posterior credible interval of the mean slope is also positive. **(C)** For predictability, in contrast, the credible interval is negative, indicating a credible decrease in predictability with hierarchy score. **(D–F)** Very similar results are obtained for the *Brain Observatory* data set. **(G)** For spontaneous activity and the correlation timescale, the posterior of the mean slope is very similar to the natural movie conditions, but variability across mice is larger. **(H)** For the information timescale, in contrast, the posterior indicates a smaller slope. **(I)** For predictability, the credible interval even contains a zero slope, indicating that predictability does not necessarily decrease along the anatomical hierarchy for spontaneous activity.
