# Supplementary material for: Signatures of hierarchical temporal processing in the mouse visual system
Source: PLoS Comput Biol. 2024 Aug 22;20(8):e1012355. doi: 10.1371/journal.pcbi.1012355 (PMC11373856; doi:10.1371/journal.pcbi.1012355)
Supplement: S15 Fig — To assess the difference in temporal processing between higher cortical areas and V1, the median timescales and predictability in higher cortical areas were modelled with an offset θhc. (A) (Top) For the correlation timescale, the 95% posterior credible interval of the mean offset μθhc across all mice is positive (black bar, red dot indicates median), indicating a credible increase in timescales for higher cortical areas. (Bottom) On the level of individual mice, posteriors indicate the same effect, but are more diverse (colors indicate different mice). In particular, for some mice the posteriors also attribute probability to zero or negative offsets, which could be either due to increased uncertainty due to the smaller sampling size, or an incomplete sampling of the areas for individual mice. (B) For the information timescale the posterior credible interval of the mean offset is also positive. (C) For predictability, in contrast, the credible interval is negative, indicating a credible decrease in predictability for higher cortical areas. (D–F) Very similar results are obtained for the Brain Observatory data set. (G) For spontaneous activity and the correlation timescale, the posterior of the mean offset is very similar to the natural movie conditions, but variability across mice is larger. (H) For the information timescale, in contrast, the posterior indicates a smaller offset. (I) For predictability the credible interval even contains a zero offset, indicating that predictability under spontaneous activity is not necessarily smaller in higher cortical areas when compared to V1. (PDF) [file pcbi.1012355.s015.pdf]

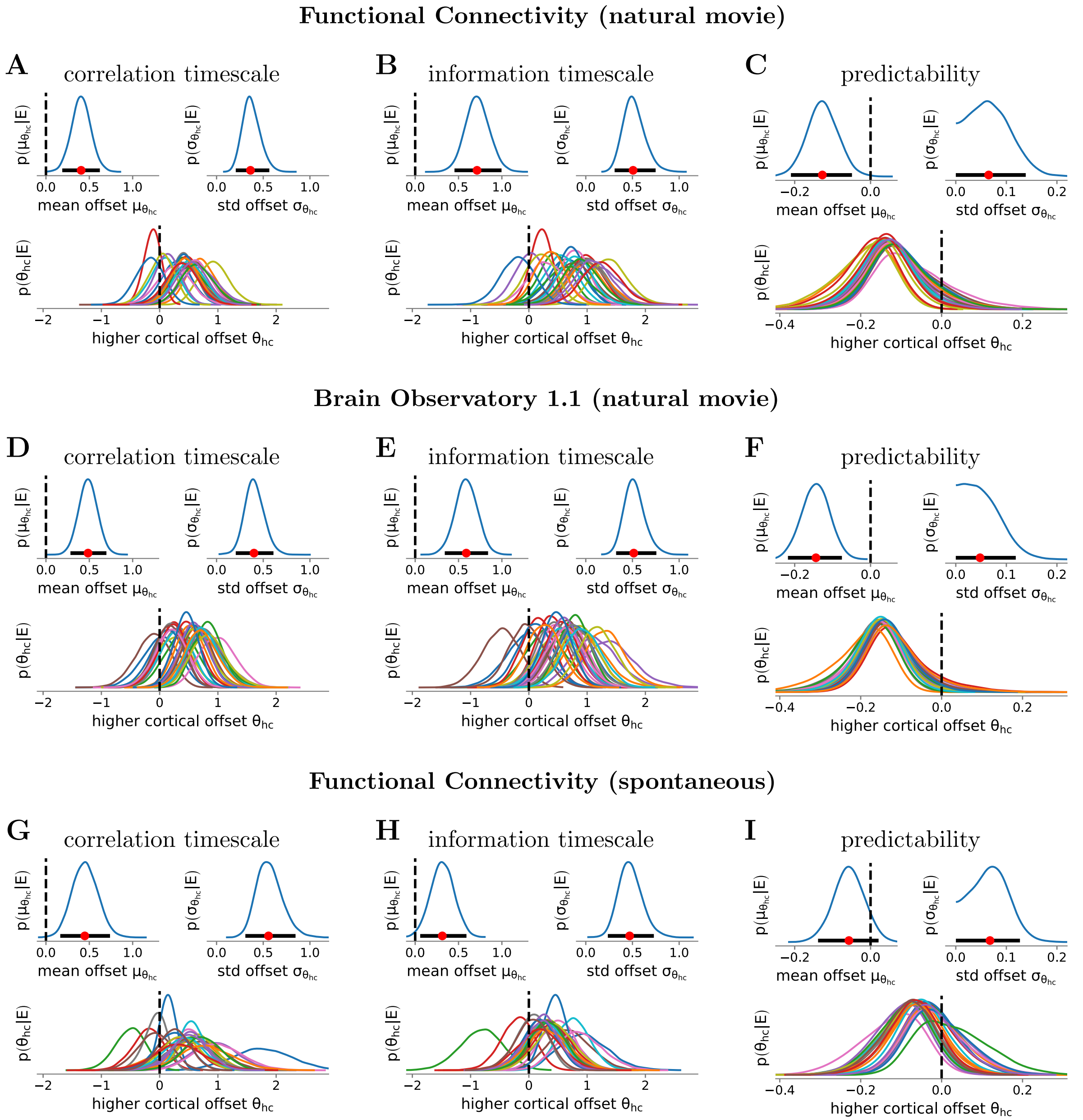

Figure S15. Posterior distributions of the higher cortical offset reveal a significant increase in timescales and decrease in predictability for higher cortical areas. To assess the difference in temporal processing between higher cortical areas and V1, the median timescales and predictability in higher cortical areas were modelled with an offset θ_{hc}. **(A)** (Top) For the correlation timescale, the 95 % posterior credible interval of the mean offset $\mu_{\theta_{\mathrm{hc}}}$ across all mice is positive (black bar, red dot indicates median), indicating a credible increase in timescales for higher cortical areas. (Bottom) On the level of individual mice, posteriors indicate the same effect, but are more diverse (colors indicate different mice). In particular, for some mice the posteriors also attribute probability to zero or negative offsets, which could be either due to increased uncertainty due to the smaller sampling size, or an incomplete sampling of the areas for individual mice. **(B)** For the information timescale the posterior credible interval of the mean offset is also positive. **(C)** For predictability, in contrast, the credible interval is negative, indicating a credible decrease in predictability for higher cortical areas. **(D–F)** Very similar results are obtained for the *Brain Observatory* data set. **(G)** For spontaneous activity and the correlation timescale, the posterior of the mean offset is very similar to the natural movie conditions, but variability across mice is larger. **(H)** For the information timescale, in contrast, the posterior indicates a smaller offset. **(I)** For predictability the credible interval even contains a zero offset, indicating that predictability under spontaneous activity is not necessarily smaller in higher cortical areas when compared to V1.
