# Supplementary material for: Signatures of hierarchical temporal processing in the mouse visual system
Source: PLoS Comput Biol. 2024 Aug 22;20(8):e1012355. doi: 10.1371/journal.pcbi.1012355 (PMC11373856; doi:10.1371/journal.pcbi.1012355)
Supplement: S16 Fig — Overall, posteriors over intercepts of the cortical groups model reveal a high diversity among mice, whereas offsets are more similar between different mice (S15 Fig). (A) (Top) Posterior distributions of the mean μθ0 and standard deviation σθ0 of the model intercept θ0 for the correlation timescale. (Bottom) Posterior distributions of θ0 for individual mice (colors indicate different mice). (B,C) Same as A, but for information timescale and predictability. (D–F) Same as A–C, but for the Brain Observatory data set. (G–I) Same as A–C, but for spontaneous activity in the Functional Connectivity data set. (I) For predictability, intercepts are much more similar between mice, because the higher cortical offset is much closer to 0 (S15 Fig), and thus may partially account for differences in median predictability between mice. For all panels red dots and black bars indicate the median and 95% highest-density-interval of the posterior distribution. (PDF) [file pcbi.1012355.s016.pdf]

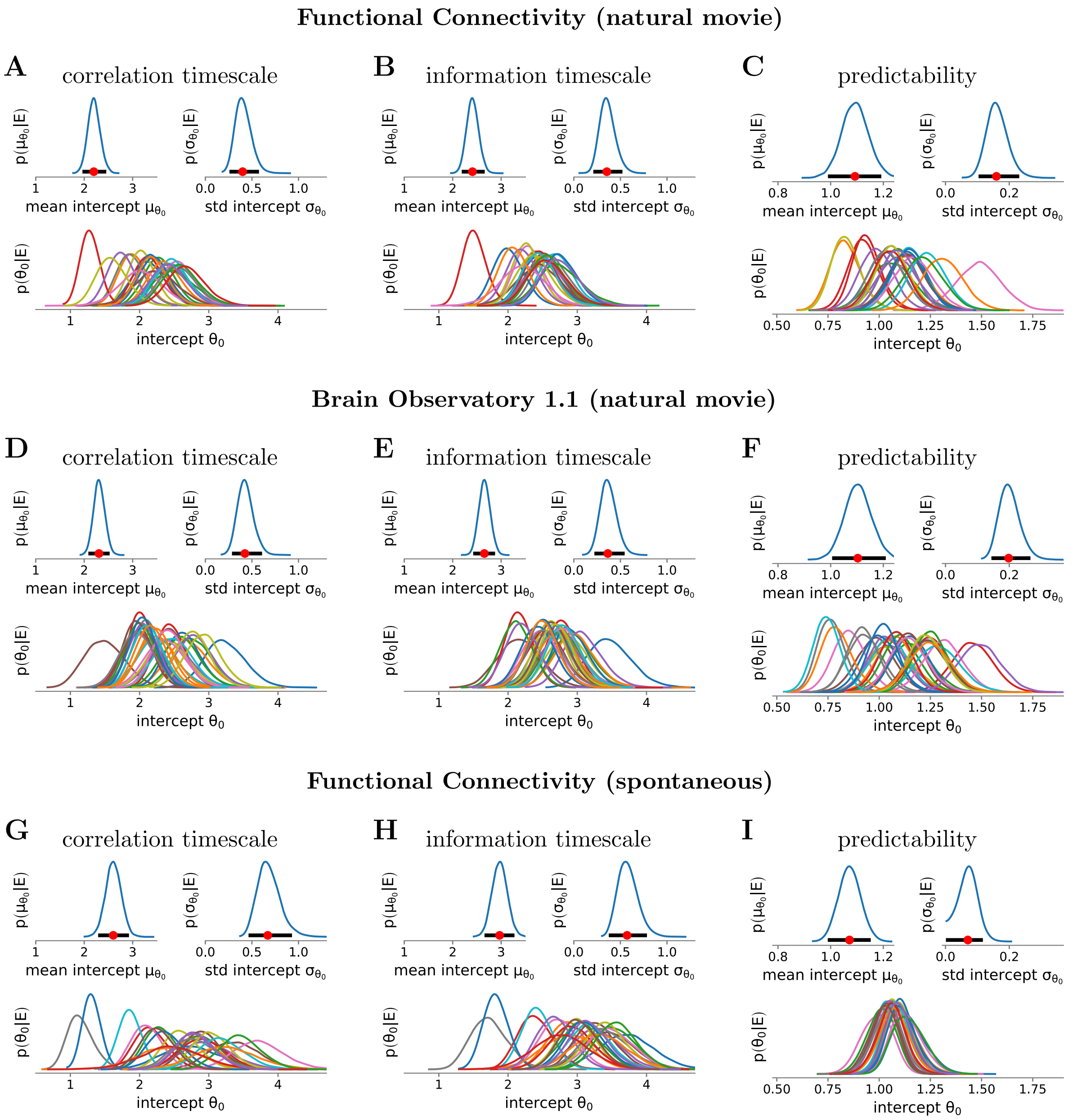

Figure S16. Posterior distributions of intercepts in the cortical groups model. Overall, posteriors over intercepts of the cortical groups model reveal a high diversity among mice, whereas offsets are more similar between different mice (Supplementary Fig. S15). **(A)** (Top) Posterior distributions of the mean μ_{θ_0} and standard deviation σ_{θ_0} of the model intercept θ_0 for the correlation timescale. (Bottom) Posterior distributions of θ_0 for individual mice (colors indicate different mice). **(B,C)** Same as A, but for information timescale and predictability. **(D–F)** Same as A–C, but for the *Brain Observatory* data set. **(G–I)** Same as A–C, but for spontaneous activity in the *Functional Connectivity* data set. **(I)** For predictability, intercepts are much more similar between mice, because the higher cortical offset is much closer to 0 (Supplementary Fig. S15), and thus may partially account for differences in median predictability between mice. For all panels red dots and black bars indicate the median and 95 % highest-density-interval of the posterior distribution.
