# Supplementary material for: Signatures of hierarchical temporal processing in the mouse visual system
Source: PLoS Comput Biol. 2024 Aug 22;20(8):e1012355. doi: 10.1371/journal.pcbi.1012355 (PMC11373856; doi:10.1371/journal.pcbi.1012355)
Supplement: S17 Fig — (A) Posterior density function of non-hierarchical parameters for the Functional Connectivity data set (red dot indicates median and black bar the 95% highest density interval). The highest density interval (HDI) for the receptive field predictor θrf is negative for the timescales for all stimulus conditions (A–C), hence units with a significant receptive field tend to have smaller timescales. In contrast, the HDI is positive for predictability for the movie conditions (A,B) and negative for spontaneous activity (C), indicating that units with a receptive field have higher predictability when driven with a stimulus with temporal correlations, in line with the idea that predictability is induced by visual stimuli. The HDI for the log firing rate predictor θlog ν is positive for the correlation timescale and negative for the information measures, thus units with higher firing rate tend to have longer correlation timescales but smaller predictability. The posterior of the shape parameter α is concentrated on negative values, indicating a negatively skewed distribution of log correlation and information timescales. (B, C) Apart from posteriors of the receptive field predictor for predictability, the posteriors for the natural movie condition in the Brain Observatory 1.1 data set (B) and spontaneous activity in the Functional Connectivity data set (C) are overall very similar to the posteriors in (A). (PDF) [file pcbi.1012355.s017.pdf]

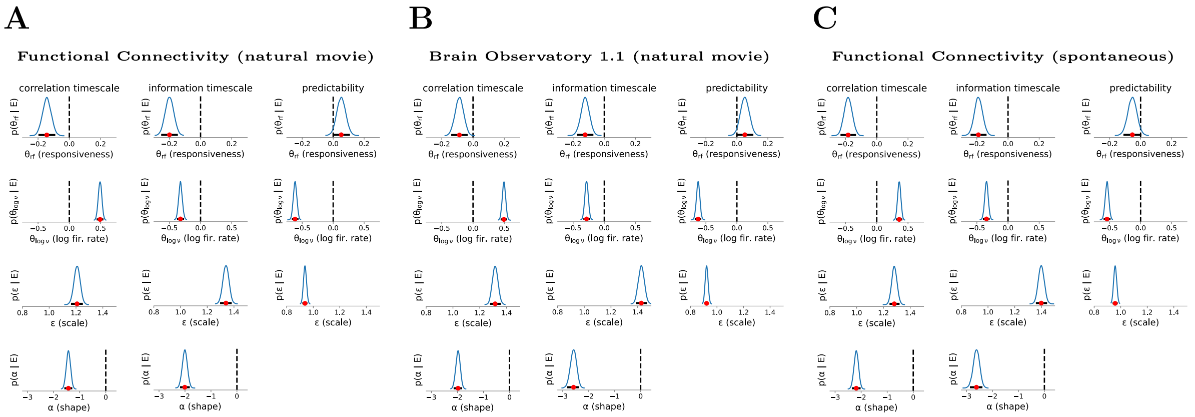

Figure S17. Posteriors for non-hierarchical parameters of the cortical hierarchy model. (A) Posterior density function of non-hierarchical parameters for the *Functional Connectivity* data set (red dot indicates median and black bar the 95% highest density interval). The highest density interval (HDI) for the receptive field predictor θ_{rf} is negative for the timescales for all stimulus conditions (A–C), hence units with a significant receptive field tend to have smaller timescales. In contrast, the HDI is positive for predictability for the movie conditions (A,B) and negative for spontaneous activity (C), indicating that units with a receptive field have higher predictability when driven with a stimulus with temporal correlations, in line with the idea that predictability is induced by visual stimuli. The HDI for the log firing rate predictor $\theta_{\log\nu}$ is positive for the correlation timescale and negative for the information measures, thus units with higher firing rate tend to have longer correlation timescales but smaller predictability. The posterior of the shape parameter α is concentrated on negative values, indicating a negatively skewed distribution of log correlation and information timescales. **(B, C)** Apart from posteriors of the receptive field predictor for predictability, the posteriors for the natural movie condition in the *Brain Observatory 1.1* data set (B) and spontaneous activity in the *Functional Connectivity* data set (C) are overall very similar to the posteriors in (A).
