# Supplementary material for: Signatures of hierarchical temporal processing in the mouse visual system
Source: PLoS Comput Biol. 2024 Aug 22;20(8):e1012355. doi: 10.1371/journal.pcbi.1012355 (PMC11373856; doi:10.1371/journal.pcbi.1012355)
Supplement: S18 Fig — Posterior densities for non-hierarchical parameters of the cortical groups model are very similar to the hierarchy score model (c.f. S17 Fig). (PDF) [file pcbi.1012355.s018.pdf]

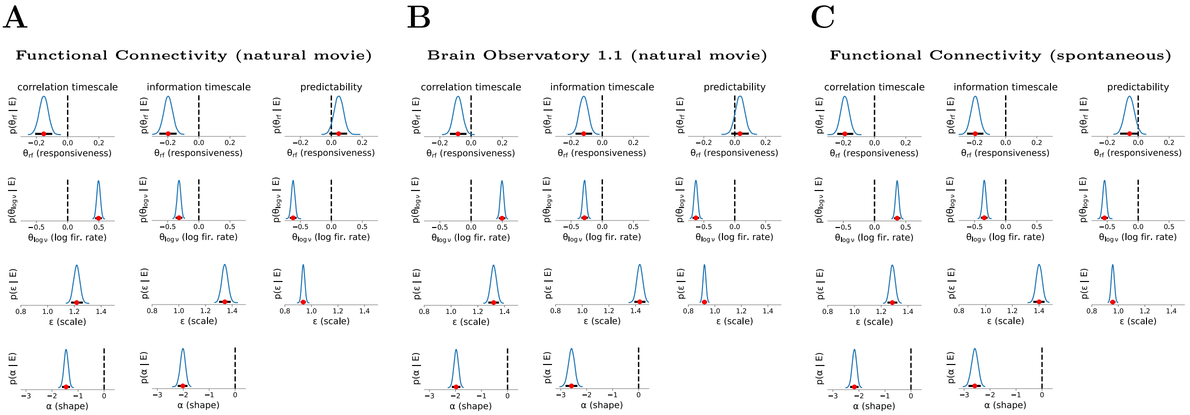

Figure S18. Posteriors for non-hierarchical parameters of the cortical groups model. Posterior densities for non-hierarchical parameters of the cortical groups model are very similar to the hierarchy score model (c.f. Supplementary Fig. S17).
