# Supplementary material for: Signatures of hierarchical temporal processing in the mouse visual system
Source: PLoS Comput Biol. 2024 Aug 22;20(8):e1012355. doi: 10.1371/journal.pcbi.1012355 (PMC11373856; doi:10.1371/journal.pcbi.1012355)
Supplement: S19 Fig — (A) To test whether the cortical hierarchy model is well calibrated, we compared the posterior predictive likelihood of the model (blue line) with the observed likelihood of timescales and predictability (black line), which overall show a good agreement. Note the skewness in the distribution of log timescales, which led us to use a skew normal distribution to model the residual variability in the timescale data. To obtain a better check for the conditional probabilities of individual data points, and not only the pooled data, we performed LOO cross-validated probability integral transform (PIT) posterior predictive checks [108]. In LOO-PIT, the model is fitted for each datum yi to all data except yi, here denoted as y−i. Pr(yimodel≤yi|y-i) then represents the probability that a value yimodel simulated from the fitted model is less or equal to yi. If the model and data distributions are the same, then the distribution of these probabilities over all data points yi (thick blue line) should be uniform [108], hence we compare it to 100 simulated data sets from a uniform distribution (thin blue lines). The model appears well calibrated for the predictability, whereas for the timescales the model tends to under-represent intermediate values, and to over-represent small and high values. (B) Same as A, but for the cortical groups model. Notably, the models appear to be equally well calibrated, hence we do not expect the differences in their predictive power to be caused by a sub-optimal calibration of the models. (PDF) [file pcbi.1012355.s019.pdf]

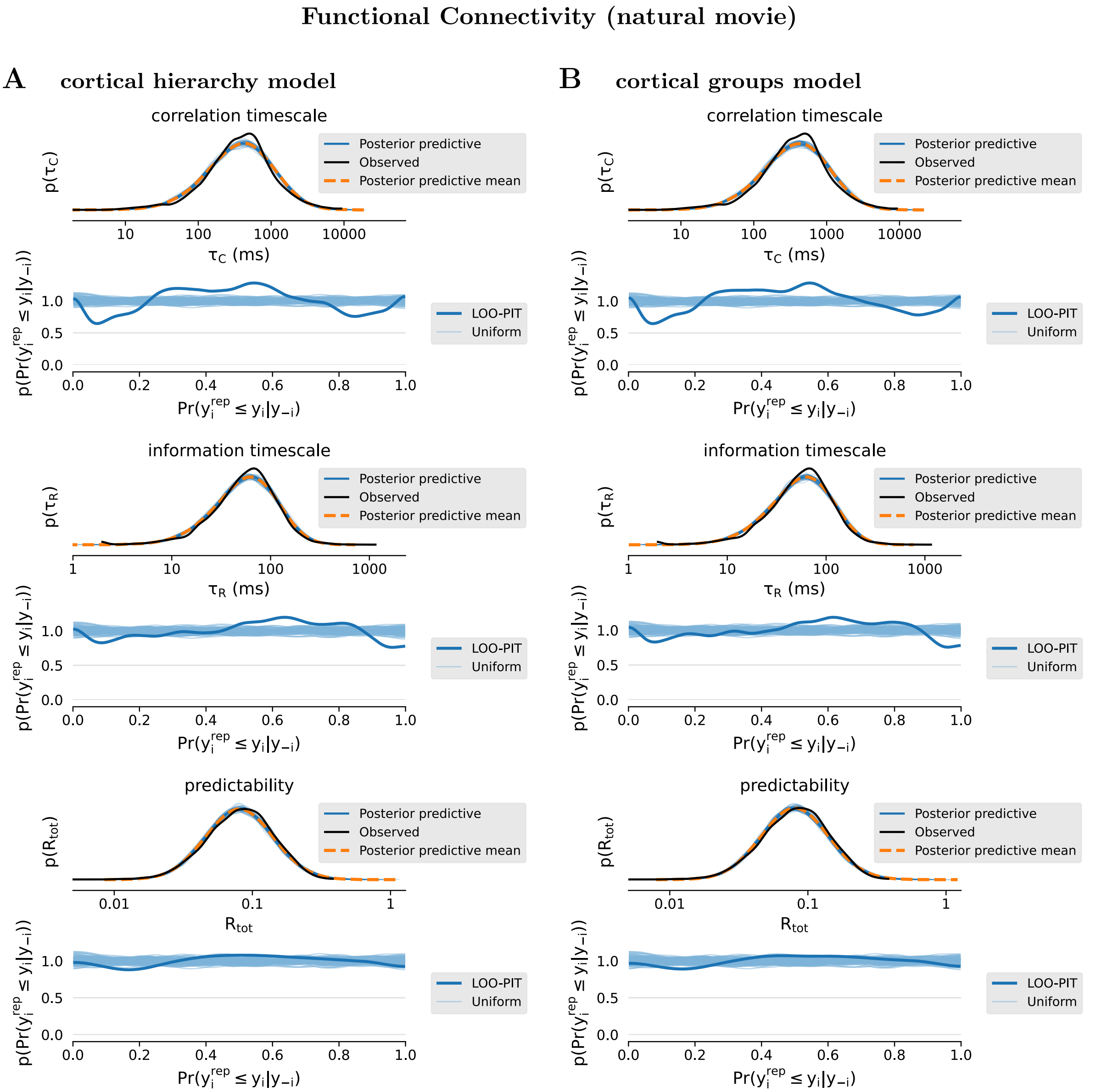

Figure S19. Posterior predictive checks of the different Bayesian models applied to the *natural movie* condition in the *Functional Connectivity* data set. (A) To test whether the cortical hierarchy model is well calibrated, we compared the posterior predictive likelihood of the model (blue line) with the observed likelihood of timescales and predictability (black line), which overall show a good agreement. Note the skewness in the distribution of log timescales, which led us to use a skew normal distribution to model the residual variability in the timescale data. To obtain a better check for the conditional probabilities of individual data points, and not only the pooled data, we performed LOO cross-validated probability integral transform (PIT) posterior predictive checks [108]. In LOO-PIT, the model is fitted for each datum y_i to all data except y_i, here denoted as $\mathbf{y}^{-i}$. $\Pr(y_i^{\text{model}} \leq y_i|\mathbf{y}^{-i})$ then represents the probability that a value y_i^{model} simulated from the fitted model is less or equal to y_i. If the model and data distributions are the same, then the distribution of these probabilities over all data points y_i (thick blue line) should be uniform [108], hence we compare it to 100 simulated data sets from a uniform distribution (thin blue lines). The model appears well calibrated for the predictability, whereas for the timescales the model tends to under-represent intermediate values, and to over-represent small and high values. **(B)** Same as A, but for the cortical groups model. Notably, the models appear to be equally well calibrated, hence we do not expect the differences in their predictive power to be caused by a sub-optimal calibration of the models.
