# Supplementary material for: Signatures of hierarchical temporal processing in the mouse visual system
Source: PLoS Comput Biol. 2024 Aug 22;20(8):e1012355. doi: 10.1371/journal.pcbi.1012355 (PMC11373856; doi:10.1371/journal.pcbi.1012355)
Supplement: S20 Fig — Same as S19 Fig, but for the natural movie condition in the Brain Observatory 1.1 data set. For these data, the model is slightly worse calibrated for the correlation timescale. (PDF) [file pcbi.1012355.s020.pdf]

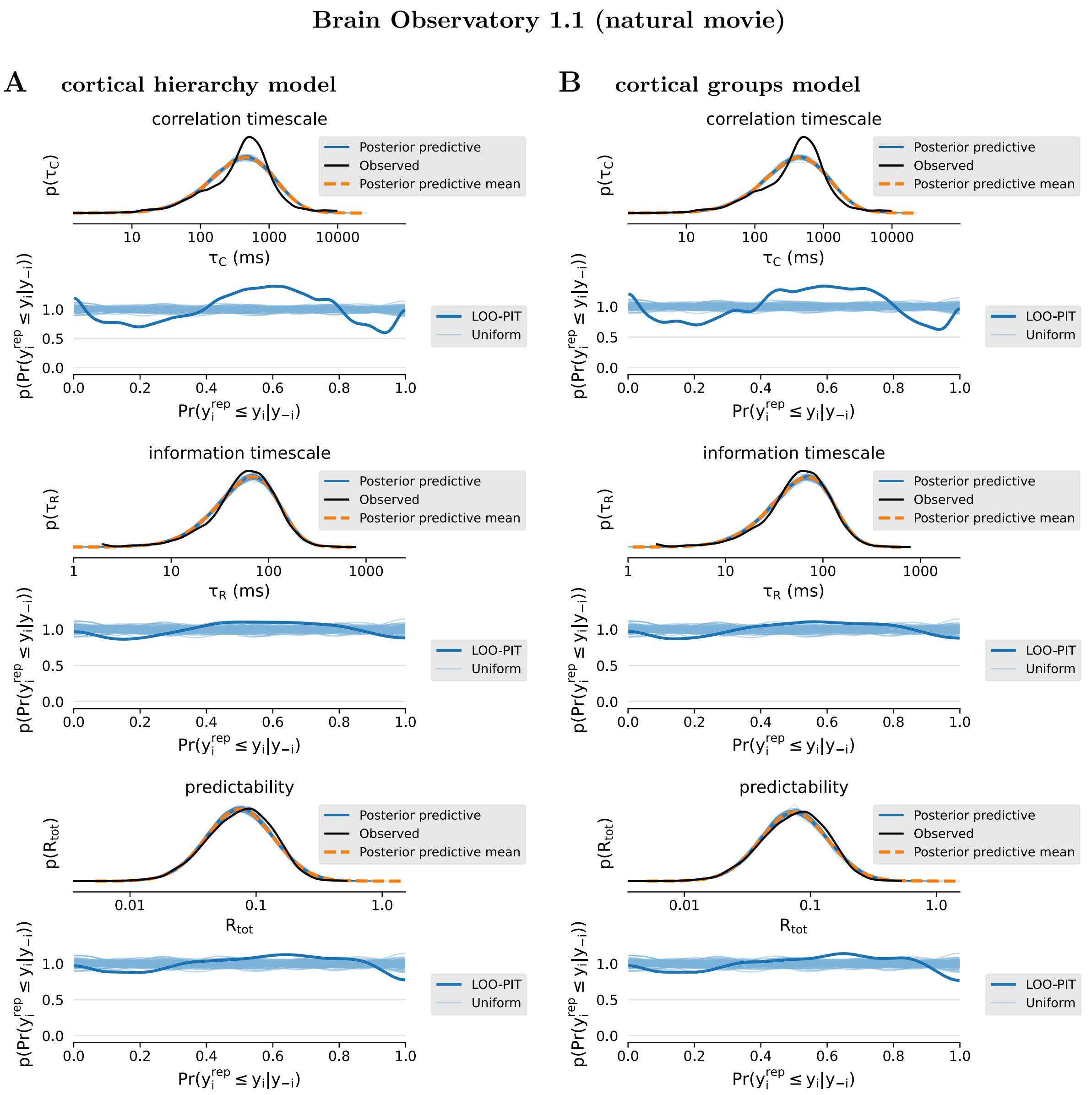

Figure S20. Posterior predictive checks of the different hierarchical models applied to the natural movie condition in the *Brain Observatory* data set. Same as Fig. S19, but for the natural movie condition in the *Brain Observatory 1.1* data set. For these data, the model is slightly worse calibrated for the correlation timescale.
