# Supplementary material for: Signatures of hierarchical temporal processing in the mouse visual system
Source: PLoS Comput Biol. 2024 Aug 22;20(8):e1012355. doi: 10.1371/journal.pcbi.1012355 (PMC11373856; doi:10.1371/journal.pcbi.1012355)
Supplement: S21 Fig — Same as S19 Fig, but for spontaneous activity in the Functional Connectivity data set. For these data, the model is slightly worse calibrated, in particular for the correlation timescale and predictability. (PDF) [file pcbi.1012355.s021.pdf]

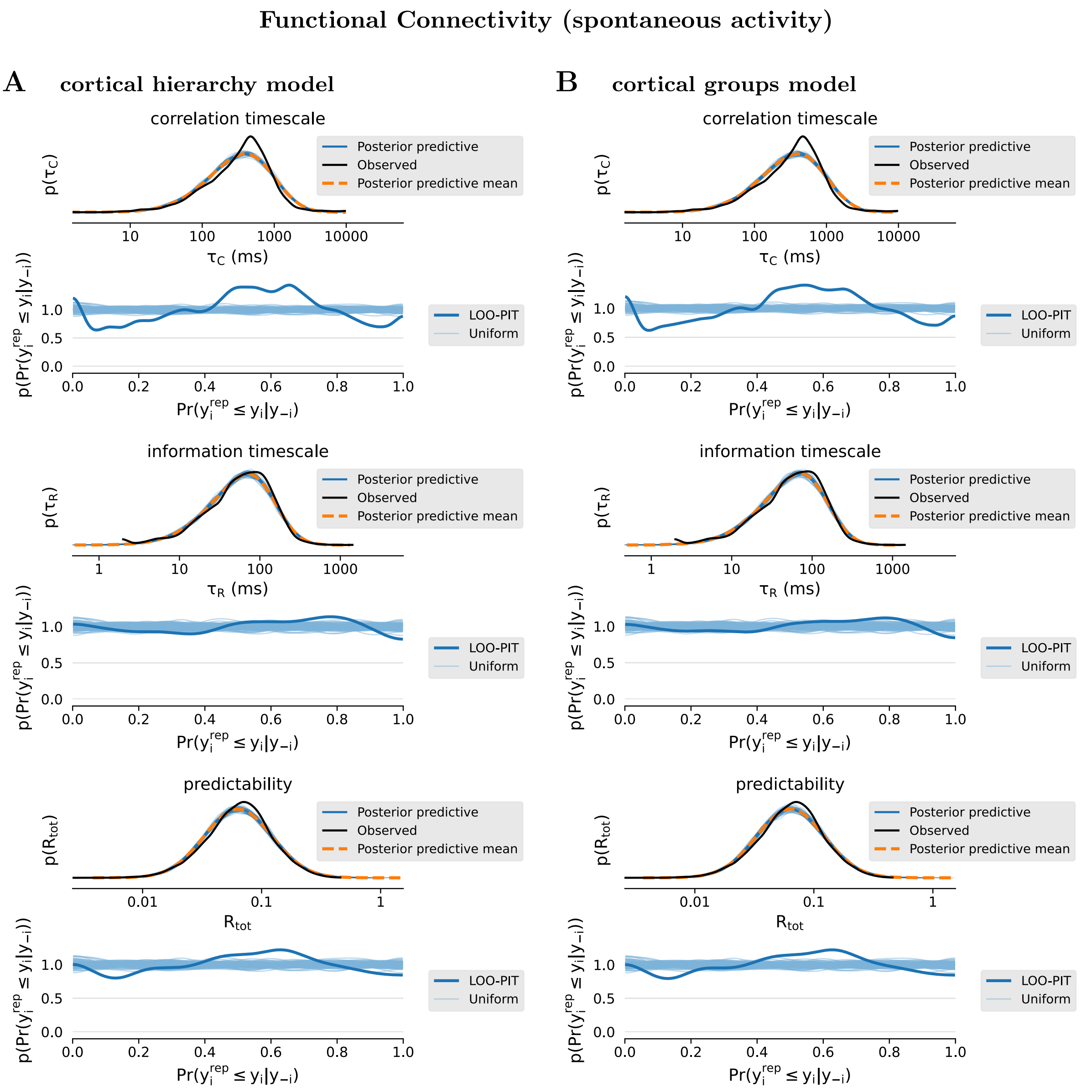

Figure S21. Posterior predictive checks of the different hierarchical models applied to *spontaneous activity* in the *Functional Connectivity* data set. Same as Fig. S19, but for spontaneous activity in the *Functional Connectivity* data set. For these data, the model is slightly worse calibrated, in particular for the correlation timescale and predictability.
