# Supplementary material for: Signatures of hierarchical temporal processing in the mouse visual system
Source: PLoS Comput Biol. 2024 Aug 22;20(8):e1012355. doi: 10.1371/journal.pcbi.1012355 (PMC11373856; doi:10.1371/journal.pcbi.1012355)
Supplement: S22 Fig — We compared across areas to determine if there was a systematic difference in timescales and predictability between stimulation with a natural movie and spontaneous activity under grey screen illumination. While median correlation timescales do not systematically differ between stimulus conditions, the median information timescale is significantly larger under spontaneous activity for most areas, whereas median predictability is significantly smaller (p-values were obtained using Wilcoxon signed-rank tests, where only significant p-values after Bonferroni correction are reported). Moreover, the relative median difference Δ between conditions is higher for predictability, indicating a stronger overall effect. (PDF) [file pcbi.1012355.s022.pdf]

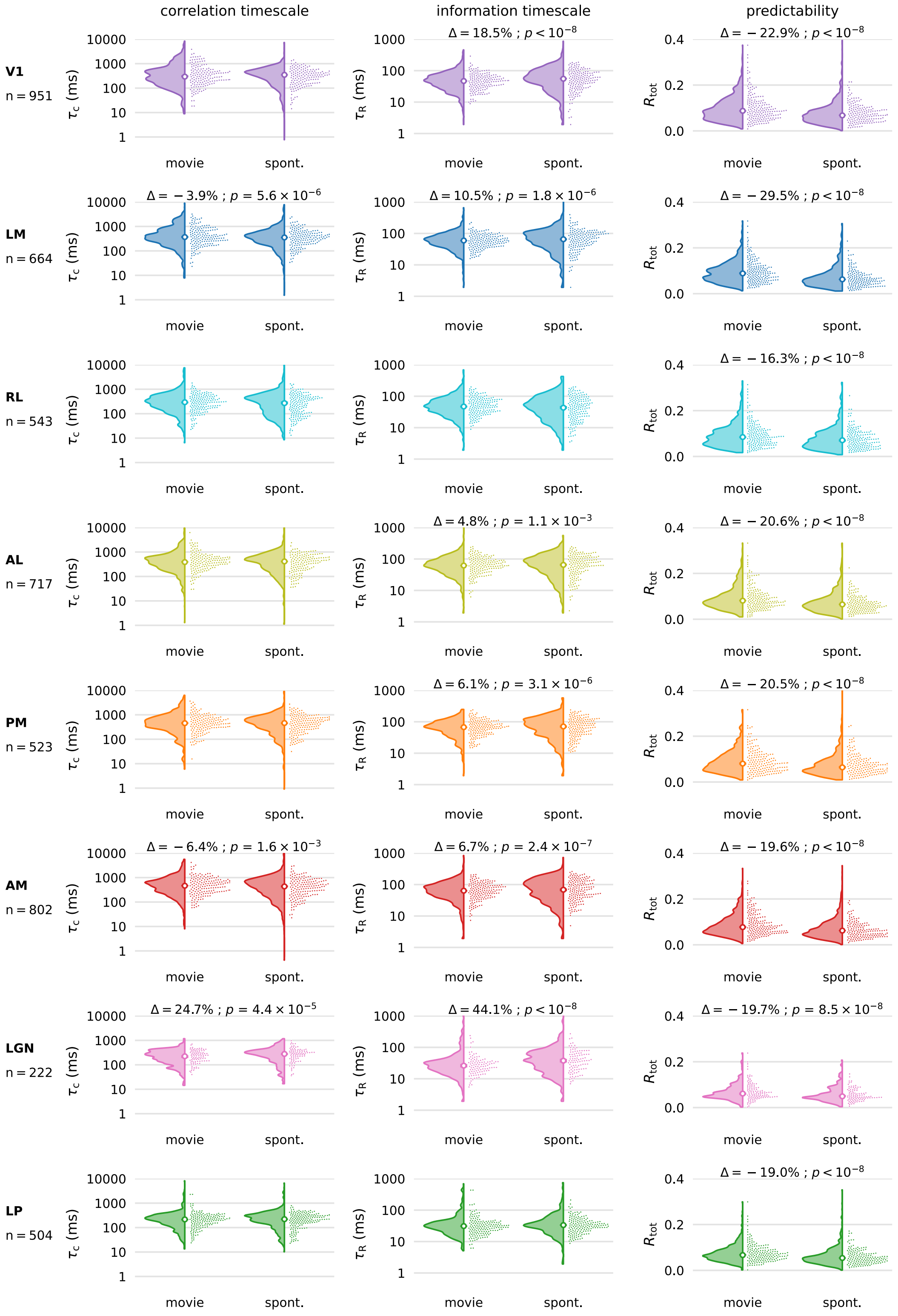

Figure S22. Area-wise comparison of timescales and predictability between natural movie and spontaneous activity in the *Functional Connectivity* data set. We compared across areas to determine if there was a systematic difference in timescales and predictability between stimulation with a natural movie and spontaneous activity under grey screen illumination. While median correlation timescales do not systematically differ between stimulus conditions, the median information timescale is significantly larger under spontaneous activity for most areas, whereas median predictability is significantly smaller (p-values were obtained using Wilcoxon signed-rank tests, where only significant p-values after Bonferroni correction are reported). Moreover, the relative median difference Δ between conditions is higher for predictability, indicating a stronger overall effect.
