# Supplementary material for: Signatures of hierarchical temporal processing in the mouse visual system
Source: PLoS Comput Biol. 2024 Aug 22;20(8):e1012355. doi: 10.1371/journal.pcbi.1012355 (PMC11373856; doi:10.1371/journal.pcbi.1012355)
Supplement: S23 Fig — For some cortical visual areas the direction selectivity of individual units (measured on drifting gratings shown in 8 different directions [32]) is negatively correlated with the correlation and information timescale and for all areas positively correlated with predictability. Dots show values for each unit and lines show the linear regression with Pearson correlation coefficient r and corresponding two sided p-value p. Regression lines are only shown for areas with significant correlations after Bonferroni multiple comparison correction. For cortical areas V1 and LM, information timescales are also negatively correlated with direction selectivity. (PDF) [file pcbi.1012355.s023.pdf]

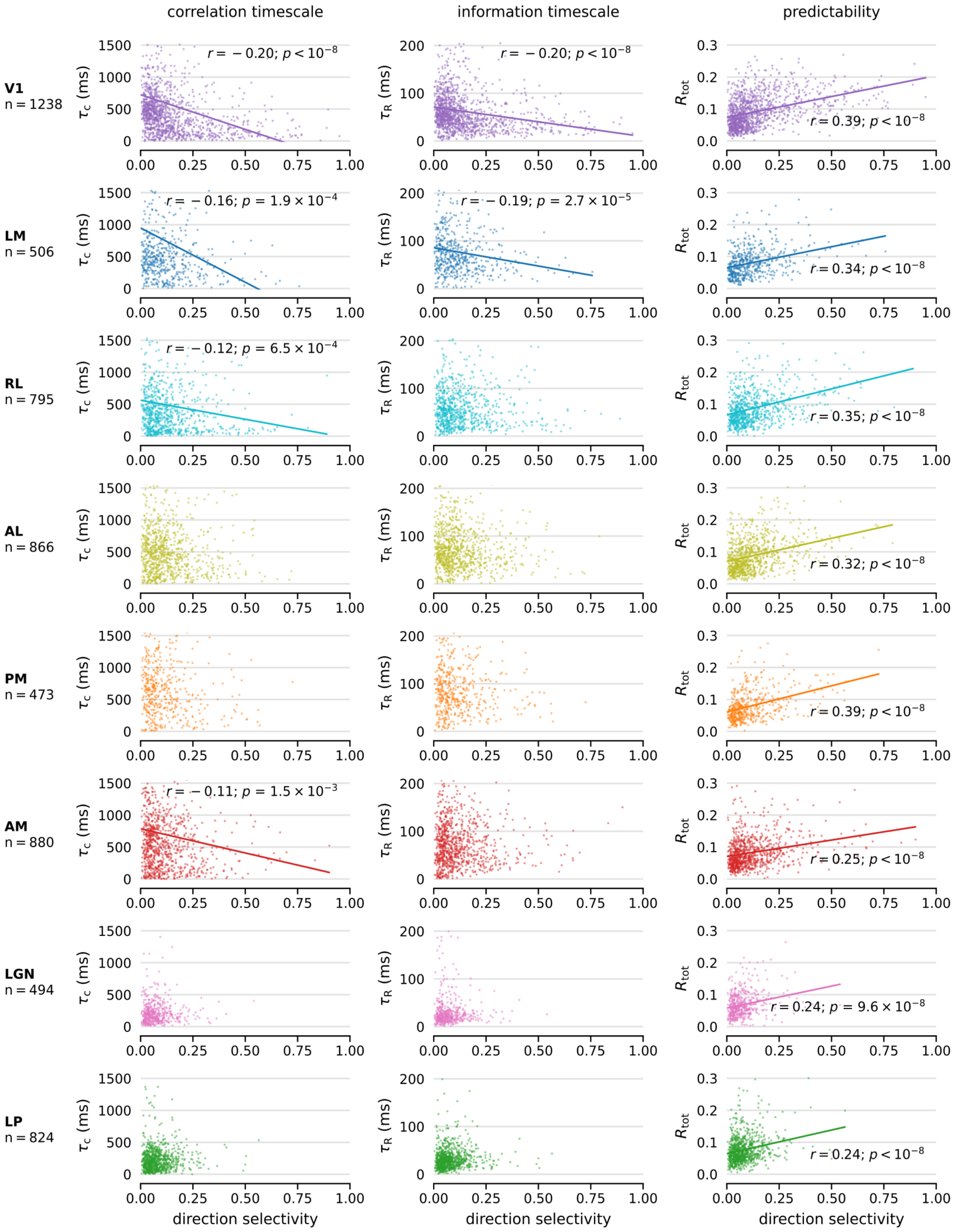

Figure S23. Relation of timescales and predictability to direction selectivity for different visual areas. For some cortical visual areas the direction selectivity of individual units (measured on drifting gratings shown in 8 different directions [32]) is negatively correlated with the correlation and information timescale and for all areas positively correlated with predictability. Dots show values for each unit and lines show the linear regression with Pearson correlation coefficient r and corresponding two sided p-value p. Regression lines are only shown for areas with significant correlations after Bonferroni multiple comparison correction.
