# Supplementary material for: Signatures of hierarchical temporal processing in the mouse visual system
Source: PLoS Comput Biol. 2024 Aug 22;20(8):e1012355. doi: 10.1371/journal.pcbi.1012355 (PMC11373856; doi:10.1371/journal.pcbi.1012355)
Supplement: S24 Fig — (A) Correlation timescales τC of individual cortical units (dots) under stimulation with a natural movie in the Brain Observatory 1.1 data set versus the image selectivity index (measured for different static images shown to the mice [32]). Timescales τC show a weak negative correlation with image selectivity index (line shows linear regression, r gives Pearson correlation coefficient with corresponding two sided p-value). (B) Information timescales τR are very weakly negatively correlated with image selectivity index. (C) In contrast, the predictability Rtot is positively correlated with the image selectivity index. (PDF) [file pcbi.1012355.s024.pdf]

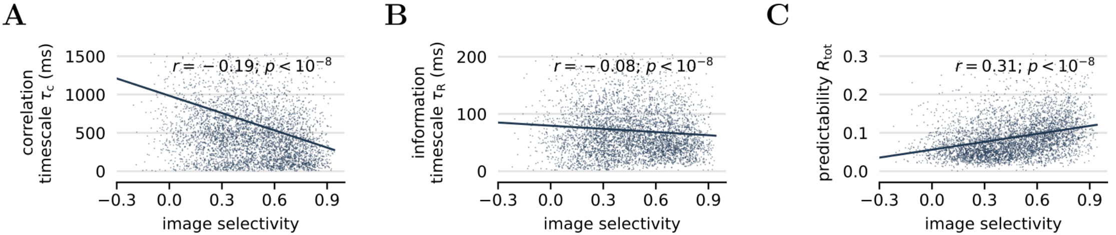

Figure S24. Relation of timescales and predictability to image selectivity of individual units for all cortical areas. (A) Correlation timescales τ_C of individual cortical units (dots) under stimulation with a natural movie in the *Brain Observatory 1.1* data set versus the image selectivity index (measured for different static images shown to the mice [32]). Timescales τ_C show a weak negative correlation with image selectivity index (line shows linear regression, r gives Pearson correlation coefficient with corresponding two sided p-value). **(B)** Information timescales τ_R are very weakly negatively correlated with image selectivity index. **(C)** In contrast, the predictability R_{tot} is positively correlated with the image selectivity index.
