# Supplementary material for: Signatures of hierarchical temporal processing in the mouse visual system
Source: PLoS Comput Biol. 2024 Aug 22;20(8):e1012355. doi: 10.1371/journal.pcbi.1012355 (PMC11373856; doi:10.1371/journal.pcbi.1012355)
Supplement: S25 Fig — For cortical visual areas the image selectivity of individual units (measured for different static images shown to the mice [32]) is negatively correlated with the correlation timescale and positively correlated with predictability. For some areas, also information timescales are negatively correlated with image selectivity. Dots show values for each unit and lines show the linear regression with Pearson correlation coefficient r and corresponding two sided p-value p. Regression lines are only shown for areas with significant correlations after Bonferroni multiple comparison correction. (PDF) [file pcbi.1012355.s025.pdf]

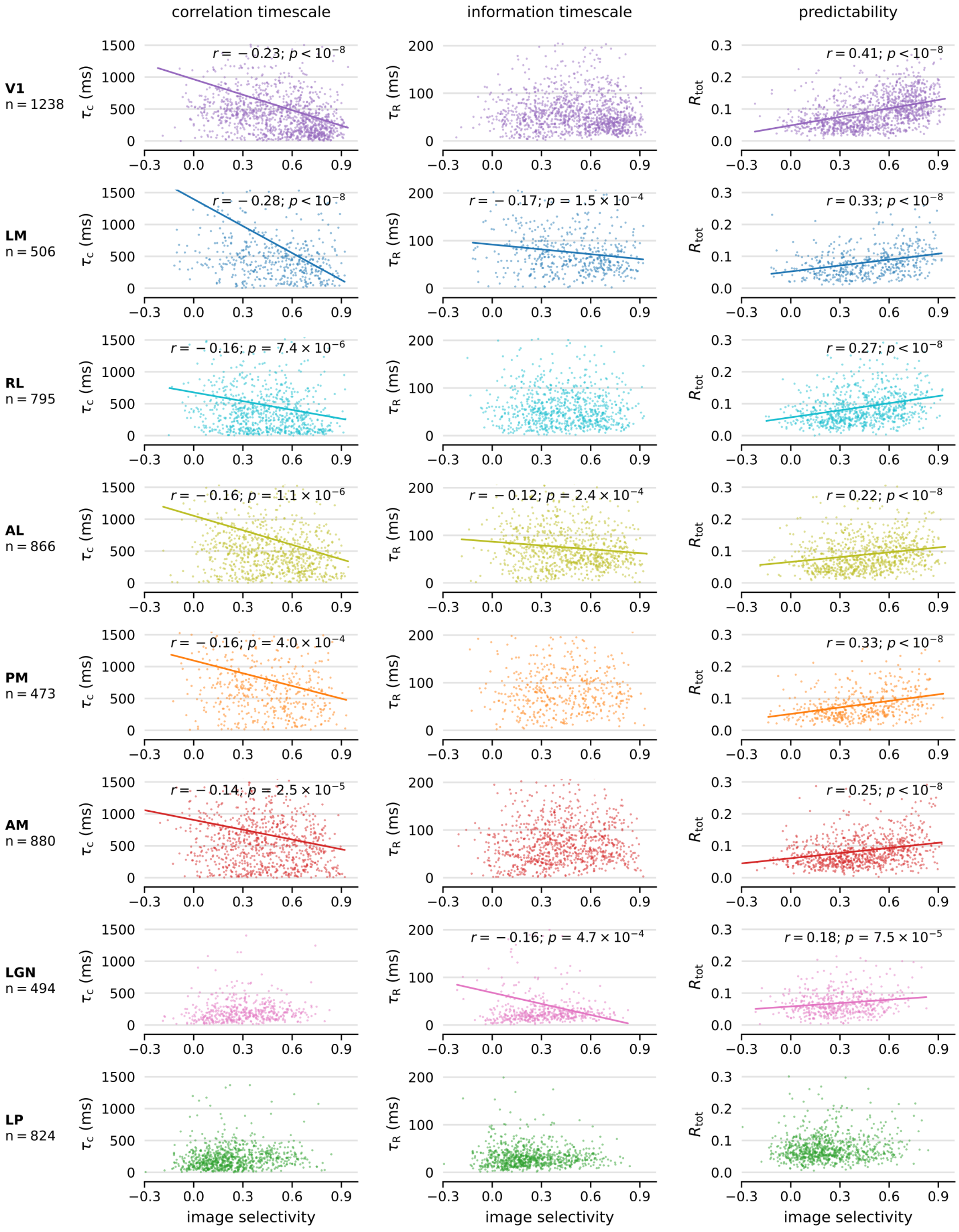

Figure S25. Relation of timescales and predictability to image selectivity for different visual areas. For cortical visual areas the image selectivity of individual units (measured for different static images shown to the mice [32]) is negatively correlated with the correlation timescale and positively correlated with predictability. For some areas, also information timescales are negatively correlated with image selectivity. Dots show values for each unit and lines show the linear regression with Pearson correlation coefficient r and corresponding two sided p-value p. Regression lines are only shown for areas with significant correlations after Bonferroni multiple comparison correction.
