# Supplementary material for: Signatures of hierarchical temporal processing in the mouse visual system
Source: PLoS Comput Biol. 2024 Aug 22;20(8):e1012355. doi: 10.1371/journal.pcbi.1012355 (PMC11373856; doi:10.1371/journal.pcbi.1012355)
Supplement: S26 Fig — To compare results from the branching network with a more realistic recurrent network, we consider an implementation of a network of N = 512 leaky integrate-and-fire neurons with synaptic plasticity on neuromorphic hardware (BrainScaleS-2) [63, 75]. In this implementation, the recurrent amplification (expressed via neural efficacy m=1-h/a) cannot be set directly, but is tuned via plasticity, which adapts to the number of input synapses kin from which each unit receives Poisson input. In particular, it has been shown that for less external input, the spike-timing-dependent plasticity tunes the network towards configurations with stronger recurrent coupling, and better integration for complex tasks [63]. To quantify the effective strength of recurrent coupling, we estimated the neural efficacy m via autoregression of the activity time series. As in the branching network, an increase in recurrence (here expressed through m, and shown for a smaller range) increases τC and τR, but decreases Rtot. Notably, in this model the source of single-neuron predictability besides recurrence is not provided through temporal correlations in the input, but by the membrane dynamics, effectively causing single-unit memory and predictability, which then gets diminished by increasing recurrence. Small dots show median values for individual network realizations, and big dots indicate median values over all network realizations for a given kin. (PDF) [file pcbi.1012355.s026.pdf]

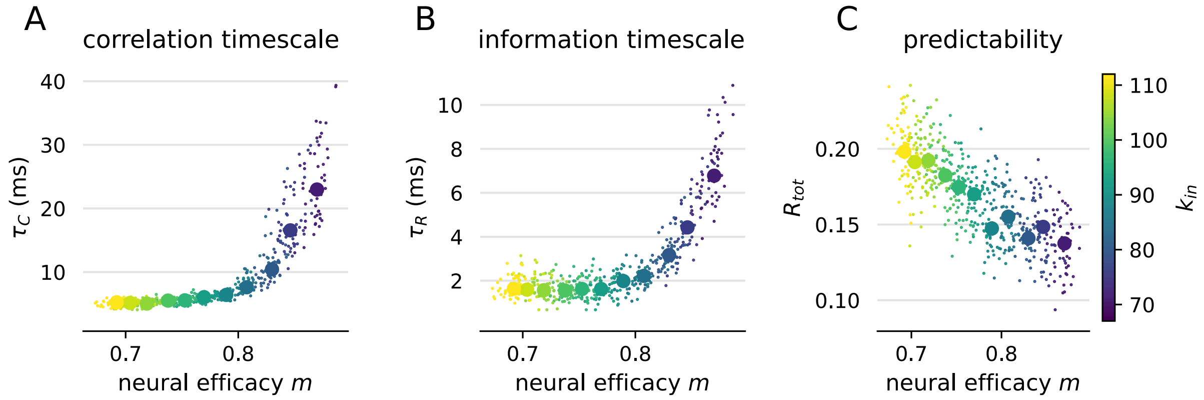


**Figure S26. Timescales and predictability in a plastic recurrent LIF network implemented on neuromorphic hardware.** To compare results from the branching network with a more realistic recurrent network, we consider an implementation of a network of $N = 512$ leaky integrate-and-fire neurons with synaptic plasticity on neuromorphic hardware (BrainScaleS-2) [63,75]. In this implementation, the recurrent amplification (expressed via neural efficacy $m = 1 - 1/a$) cannot be set directly, but is tuned via plasticity, which adapts to the number of input synapses $k_{in}$ from which each unit receives Poisson input. In particular, it has been shown that for less external input, the spike-timing-dependent plasticity tunes the network towards configurations with stronger recurrent coupling, and better integration for complex tasks [63]. To quantify the effective strength of recurrent coupling, we estimated the neural efficacy $m$ via autoregression of the activity time series. As in the branching network, an increase in recurrence (here expressed through $m$, and shown for a smaller range) increases $\tau_C$ and $\tau_R$, but decreases $R_{\mathrm{tot}}$. Notably, in this model the source of single-neuron predictability besides recurrence is not provided through temporal correlations in the input, but by the membrane dynamics, effectively causing single-unit memory and predictability, which then gets diminished by increasing recurrence. Small dots show median values for individual network realizations, and big dots indicate median values over all network realizations for a given $k_{in}$.
