# Supplementary material for: Signatures of hierarchical temporal processing in the mouse visual system
Source: PLoS Comput Biol. 2024 Aug 22;20(8):e1012355. doi: 10.1371/journal.pcbi.1012355 (PMC11373856; doi:10.1371/journal.pcbi.1012355)
Supplement: Attachment — Submitted filename: Review.pdf [file pcbi.1012355.s027.pdf]

Review

In this manuscript, the authors quantified the timescale of neural activity in mouse visual cortex using two metrics: decaying time of single-neuron autocorrelation function and an information-theoretic metric, the *predictability,* which yields both a timescale and an asymptotic value. They found a clear increase in both timescales when moving from thalamic regions to primary visual cortex and further up along the higher cortical areas. The total predictability rose from thalamus to visual cortex, but *decreased* along the visual hierarchy (as measured by an anatomical score). Importantly, Bayesian modeling showed that the within-visual-cortex timescale trends were robust and significant and a two-stage (V1 - higher areas) model did a worse job at reproducing the data then a (more-complex) fully hierarchical model. This was not true for overall predictability. In addition, the authors found a very similar hierarchical increase of time scales when analyzing spontaneous activity (in absence of visual stimuli). Finally, the authors introduce a conceptual model to show how the co-existence of sufficient recurrent activity amplification (a network effect) and single-neuron predictability source can *qualitatively* reproduce the hierarchical increase in timescales and decrease in overall predictability.

Overall, I found the paper very interesting, with some innovative and imaginative analyses (like the mutual info predictability measure) that, I believe, will turn out to be very useful in the field. Another innovative element of the study is the comparison of time scales with and without visual stimulation. The fact that similar trends were obtained in the two conditions is far from obvious and, as the authors concluded, does suggest a dominant effect of intrinsic, recurrent processing, at least under some conditions.

At the same time, I found one major issue in the study, which is the mismatch between what the authors claimed to have computed (i.e., INTRINSIC timescales) and what they have actually computed, which are metrics that depend on both intrinsic and stimulus-driven processes, as far as I can understand. This semantic mismatch does requires, in my opinion, a revision of some of the statements and conclusions of the manuscript. In addition, I believe that a more direct comparison with similar work performed by previous studies would help putting the results of the authors in a broader context and clarify some of their interpretation. Full disclosure here: I am Davide Zoccolan, the author of a study (Piasini et al 2021) that addressed very similar questions to those addressed by the authors. I prefer to disclose my name, since many of the criticisms and suggestions I have listed below derive from my very direct experience in dealing with similar data and questions. So, I believe that all my comments will be more understandable if the authors know where they come from.

MAJOR POINTS

1) As I mentioned above, the major issue I encountered in reading the manuscript is the way the authors defined the intrinsic timescales. This issue is strictly related to the way the authors formatted the Allen data. So, I refer here to the Methods sections named “Experimental data sets” and “Estimation of intrinsic timescale”.

The first aspect of the methods that is not fully clear to me is how the authors handled the neuronal responses collected across repeated presentations of the same movie. Take for instance natural_movie_one_more_repeats. As the authors wrote, this portion of the dataset contains 15 min of neuronal responses to the repeated presentation of a 30 second naturalistic movie. How are the authors dealing with such repeated presentations? Are they simply ignoring them, treating, de facto, the 15 min of recording as a unique trial of a single movie lasting many minutes (even if, in fact, the movie is made of many repeated, identical sub-movies)? Or do the authors instead extract each single presentation of the 30 sec clip to create a dataset where they look at the trial-by-trial variability of the neuronal response to this 30 sec clip, as well as compute the trial-average firing rate during the presentation of the 30 sec clip? At some point, the authors write “natural movie blocks were concatenated to then yield a total recording length of 18 minutes.” Based on this sentence, it would seem to me that the authors processed the data in the first way, i.e., as a unique, 18 min long movie (not considering the periodic nature of the actual, repeated stimulus).

This is important to understand, because, if so, this means that all the analyses of the study are performed at the single trial level (ignoring the statistical variability of the response across repeated trials). This seems consistent with their definition of the autocorrelation function from which they extracted the intrinsic time scale. In eq. 1, they simple refer to the binary activations a_t of the neurons, without mentioning whether they are computed across

repeated presentations/trials of the movie clip. The average seems to be computed across time only and not across trials.

This way to compute autocorrelation is fully legitimate of course, but it should be well understood what it implies in terms of the underlying neuronal process. For instance, the authors refer to the resulting time scale as “INTRINSIC”, but this is not what most authors have referred to as “intrinsic time scale” in previous studies. Intrinsic time scale is the one that is computed by measuring the correlation coefficient between the responses fired across different trials/repetitions of the same stimulus in different time bins during the recording. This is well defined, for instance, in (Murray et al., 2014), which calls it “the across-trial correlation between spike counts”. As explained by Murray, this autocorrelation “corrects for nonstationarity in the mean firing rate during the foreperiod (for example, ramping) because covariance and variance subtract the mean spike count for each time bin”. This means that this way of computing the autocorrelation is basically independent from the actual magnitude of the firing rate - e.g., it is independent from the stimulus-driven component of the response. It does only depend on the stochastic fluctuations of the response around its mean across different trials. In this sense it is “intrinsic” - it measures how likely it is that a cell, having fired, at a given time, during a given trial, above its across-trials average, will keep doing so, at following times during the same trial. It is a measure of “inertia” of the firing rate, which should be largely independent from the underlying stimulus producing the response. It is equivalent to the noise correlation typically computed between pairs of neurons – only, this is a noise autocorrelation computed for the same neuron across different times.

We explained even more explicitly the different nature of intrinsic and stimulus-driven (or response) time scales in (Piasini et al., 2021), where we have used the same approach of Murray to compute the intrinsic time scales, but we have also computed the stimulus driven time scale by measuring the autocorrelation function of the trial-averaged firing rate (i.e., of the PSTH of the response). In the latter, being computed over a trial-averaged response, the intrinsic dynamic of trial-by-trial fluctuations is lost. This yields a pure measure of the stimulus-induced autocorrelation of the response over time.

Now, if I have well understood what the authors did, their autocorrelation is neither purely intrinsic, nor purely stimulus driven. It is a mixture of the two. Being computed on a single trial, it does reflect the stability of the response in encoding the stimulus. At the same time, it also reflects the intrinsic noise auto-correlation. Again, nothing wrong with this, provided that the authors properly explain what their autocorrelation means and they name it properly. In fact, the resulting time scale cannot possibly be called “intrinsic”. That would be quite misleading and incorrect with respected to the definitions used in the previous literature and the meaning itself of what is measured.

As such, I believe that the authors should revise all instances where they refer to this measure as “intrinsic time scale” and rename it. Not sure what a good name can be, perhaps “within-trial, response timescale”? I want to point out that all these arguments apply also to the information time scale the authors extract from their predictability measure. Since, also in this case, predictability is computed from a minutes-long single trial (ignoring the trial-by-trial variability of the response), the resulting metric (and its time scale) depend both on the component of the response that is stimulus-determined and by the noise autocorrelation (i.e., the intrinsic dynamic) of the response. I believe the authors should be well aware of this, when they interpret their findings on the hierarchy of time scales.

2) Lines 160-162 “In contrast, median predictability decreased ($r_P = -0.93$), which might reflect an increase of adaptation that attenuates responses to predictable stimuli for an efficient code”. Also at lines 227-229: “This also suggests that the decrease in predictability along the hierarchy might reflect an active cancellation of predictability in visual stimuli, e.g., through stronger adaptation in these areas.”

The result about the hierarchical increase of information time scales and decrease of predictability is very intriguing. Here and in the Discussion, the authors propose an interpretation of this finding in terms of adaptation, and/or predictive/efficient coding. This is certainly a valid hypothesis, but other (simpler) hypotheses should also be taken into account (perhaps in the Discussion).

In particular, I believe that the information theoretic analysis performed by the authors tap onto the same mechanisms/properties we have explored in (Piasini et al., 2021) with a linear decoding analysis (see Fig. 6 of our study). The two approaches are not identical of course, but I think they basically show the same phenomenon. In their analysis, the authors measure how well past activity can predict the current one. They see that the deeper they

go along the hierarchy, the longer is the dependency from past activity, but the less is the info provided by past activity in terms of magnitude. In our analysis, we trained a linear decoder at time t_0 and then we tested how well that decoder was able to discriminate movie segments at following times. We found that, in terms of magnitude, the accuracy was larger in lower visual areas, but in terms of stability of the accuracy, this was much larger in deeper areas. This seems to be consistent (if no equivalent) to what found by the authors with their analysis. Early neuronal representations seem to contain more information about the visual input and this information, over short time scales, remain stable enough to allow, in our case, good generalization of decoding accuracy, and, in their case, good predictability. Higher areas contain less stimulus info, but, whatever they are able to represent, it remains way more stable over time.

Why this happens? The interpretation we gave of this finding in (Piasini et al., 2021) is that this is due to the much higher sensitivity of neurons in early visual areas to low-level visual features (like contrast, luminance, position, etc.), as compared to higher-order neurons. We have shown this in a previous study based on info theory (Tafazoli et al., 2017). The hypothesis we put forward in (Piasini et al., 2021) was that these low-level features remain quite informative about the stimulus (e.g., object identity) over short time scales, but they rapidly become poorly informative over long time scales. By contrast, in deep areas, most of the low-level info has been pruned - see again (Tafazoli et al., 2017), but also (Muratore et al., 2022). So, these representations are less able to discriminate the visual input (in terms of magnitude of the decoding accuracy or of the mutual info between stimulus and response) and, at short time lags, the discrimination accuracy they afford is going to be lower than in lower areas. But these higher areas encode more invariantly higher-order features and, because of this, they are able to maintain their discriminatory power very stable over time.

So, I believe that the analysis of the authors is quite consistent with this finding and interpretation of our previous study. And I believe it would be worth mentioning it as an alternative explanation of the concomitant increase of information time scale and decrease of predictability magnitude.

3) Lines 225-226. “Together, these results imply that the hierarchy in timescales seems to rely mostly on network-intrinsic mechanisms (independent of stimulus conditions or visual coding properties)”.

The result on the spontaneous activity is very interesting and, to some extent, surprising. Especially the fact that the hierarchy of temporal scales is also present in that condition. But I believe that the interpretation of this finding is more nuanced and complex than what stated here and in the Discussion. As I mentioned, the authors did not compute intrinsic time scales, but the time scale of the response resulting from a combination of intrinsic dynamics (noise autocorrelations) and stimulus induced dynamics. In (Piasini et al., 2021), we have shown that, in the Allen data, both time scales (when measured separately) increase hierarchically. In the test of the authors with spontaneous activity, there is no dynamic stimulus, so, although their metric is not, in general, a pure measure of intrinsic dynamics, we can safely assume that, under absence of stimulation, it mainly reflects intrinsic dynamics. That is, the increase of time scales across areas must be due, in this case, purely to noise autocorrelations. In such a condition, I would expect to still see the hierarchical trend, but perhaps weaker as compared to the case where a dynamic stimulus is present (because one of the two component that sets the time scale of the dynamics is gone - the stimulus-driven one). I guess I can see a hint of this by comparing the trend of Fig. 2 and Supp Fig. 12. In Fig. 2D, for instance, the range of time scales spanned by the areas is between 250 and 450 ms, while in Fig. S12D is from 300 to about 400 ms. It would be interesting if the authors could statistically test this question: is the hierarchal increase becoming less sharp in the spontaneous activity? Can the authors apply their Bayesian analysis to explicitly test this? Just as a reference: in our study we did observe, for the rat dataset, that intrinsic dynamics did add further stability to the one already afforded by the progressively invariant encoding of shape information - see Fig. 6D of (Piasini et al., 2021).

4) Discussion section. In general, I believe that the authors should revise their Discussion to consider more alternative hypotheses (see my comments above), and to make sure they do not misinterpret or overinterpret their findings, in the light of the fact that the timescales they have measured are not purely due to intrinsic processes. More specifically, I report below a few sections that, I believe, require the attention of the authors.

Lines 286-289: “We concluded that the gradients in timescales are therefore likely caused by intrinsic mechanisms, like recurrent coupling that could serve an enhanced temporal integration for higher areas [1, 15, 25, 26, 41], whereas a reduction of predictability might be the result of stronger cancellation of redundant, predictable stimuli, in line with hierarchical predictive and efficient coding”. I believe that this statement is too strong, in the light of the fact that what the authors measured is not about pure intrinsic dynamics and in the light of our findings with both rats and mice, where we did find a hierarchal increase of timescales also for the trial-averaged responses, where there is no contribution from intrinsic dynamics (Piasini et al., 2021).

Lines 381-387: “… Here, we found that timescales hardly change with visual stimulation and stimulus selectivity of the units (Fig. 3), …This is also consistent with findings from rat visual cortex [40], …, these results suggest that long intrinsic and information timescales rely primarily on properties of the network, e.g., recurrence, rather than temporal properties of the inputs”.

Again, the authors need to be careful here with this interpretation. Their arguments rest on the assumption that they have measured pure intrinsic time scales, which is not the case. In addition, the authors seem to conclude that the timescale they measured are stimulus-independent, so largely to due to recurrent connectivity, because of their findings with spontaneous activity and because they “timescales hardly change with stimulus selectivity of the units”. However, the latter argument is weak in my opinion, because the most important tuning property that should have been assessed in order to support this claim is whether the recorded cells were more simple-like (i.e., phase sensitive) or more complex-like (i.e., more phase invariant). The former would be expected to show faster time scales than the latter, because of the larger translation/phase-invariance of the latter. We did find this distinction in V1 [see (Matteucci and Zoccolan, 2020), Fig. 4 and 5]. Since the authors have not measured this property, it is unclear what to expect from their analysis - I mean, there is no clear prediction, as far as I can think, about whether timescales should depend in some way or another from direction selectivity.

Lines 388-389: “Of particular interest from a coding perspective are our results on predictability Rtot, because they imply that the temporal redundancy in spike trains decreases along the hierarchy”. And also lines 414-416: “In contrast, the stimulus-dependent decrease in predictability is in agreement with efficient coding as well as an observed increase of adaptation in rodents, and might constitute another hallmark of hierarchical temporal processing”. These statements are too strong. I believe that here it should be discussed the alternative hypothesis I mentioned at point 2. That is, it could be the higher sensitivity of early visual areas to low level features (i.e., the richer, more faithful representation of visual images) to allow Rtot to be larger in these areas and then to gradually decrease in higher order areas. This would be a purely feedforward mechanism that does not involve increased adaptation or predictive signals across the hierarchy.

MINOR POINTS

1) Lines 61-63. “there seems to be no clear global processing hierarchy from sensory to transmodal areas [29], as more of mouse cortex is used for sensory processing of specific modalities, instead of integrating information across modalities”.

I only partially agree with the first part of the sentence: functional evidence of hierarchical processing is indeed weak in the mouse. E.g., see some recent modeling studies of neuronal representations of natural images along the mouse visual system, where the hierarchy seems quite shallow (Cadena et al., 2019; Nayebi et al., 2023). At the same time, some other studies (like the Allen paper itself from which the authors took the data) suggest the existence of a quite clear hierarchy in terms of a number of basic tuning and response properties. In addition, at the anatomical level, the evidence of a hierarchy is quite substantial, and suggests even the presence of a ventral and a dorsal

pathway. In addition to the Allen studies, I suggest looking at the work of Burkhalter's lab or to a few reviews on the topic (Niell, 2011; Glickfeld et al., 2014; Glickfeld and Olsen, 2017). So, the authors should be a bit more nuanced in their statement here and refer to a wider pool of studies to support their statement.

Also, I disagree with the second part of the sentence: "as more of mouse cortex is used for sensory processing of specific modalities, instead of integrating information across modalities". If anything, sensory cortices in rodents are more multimodal than in primates. E.g., V1, A1 and S1 are coupled by horizontal cortico-cortical projections that are not quite present in monkeys. Se for instance: (Iurilli et al., 2012; Ibrahim et al., 2016; Meijer et al., 2017; Deneux et al., 2019; Oude Lohuis et al., 2024). It is true that we do not quite understand the role played by this cross-modal inputs to primary sensory cortices, but for sure it is not correct to state that mouse cortex is very unimodal.

2) Lines 192-196. "For rodents, a prominent hypothesis is that feedforward processing leads to more persistent stimulus representations for higher areas [40], thereby causing the longer timescales under visual stimulation. An alternative hypothesis is that stronger recurrence enables longer integration of visual information, similar to what is thought to cause longer timescales in primates".

The way in which this paragraph is written is a bit misleading. The authors seem to claim that the "feedforward" hypothesis is specific to the rodent visual system, while the "recurrent" hypothesis stems from the primate literature. However, at least insofar the "feedforward" hypothesis is concerned, the opposite is true – this hypothesis stems from what it is known about the monkey ventral stream at both the empirical and theoretical level. Empirically, the increase of invariance to transformations of static images should result in an increased temporal stability of the representations, when dynamic stimuli are used. From a computational point of view, learning of transformation-invariant representations is thought to be mediated by unsupervised adaptation to the temporal continuity of the visual input (the so-called unsupervised temporal learning), thus resulting, again, in progressively slower representations along the ventral stream – see (Matteucci et al., 2024) for a review. The problem is that this hypothesis has never been quite systematically tested along the monkey visual stream. The fact that, in (Piasini et al., 2021), we managed to test it in the rat homologue of the ventral stream, does not mean, however, that the hypothesis was actually born in the rodent literature. So, the way in which this hypothesis is presented here should be corrected, giving the proper credit to the monkey ventral stream literature and the computational models inspired by this literature.

3) Lines 328-330: "whereas latero- and posteromedial areas LM and PM are thought to play a stronger role in object perception, analogous to the dorsal and ventral streams described in other species". Here, attention should be paid not to confound putative ventral and dorsal stream areas of the mouse cortex. LM, along with LI, P and POR are thought to be part of the mouse ventral stream. Instead, PM, along with AM, A, AL and RL are thought to be part of the dorsal stream – see (Niell, 2011; Glickfeld et al., 2014; Glickfeld and Olsen, 2017) and the work (already cited by the authors) of Burkhalter's lab.

REFERENCES

Cadena SA, Sinz FH, Muhammad T, Froudarakis E, Cobos E, Walker EY, Reimer J, Bethge M, Tolias A, Ecker AS (2019) How well do deep neural networks trained on object recognition characterize the mouse visual system? In NeurIPS Neuro AI Workshop .

Deneux T, Harrell ER, Kempf A, Ceballo S, Filipchuk A, Bathellier B (2019) Context-dependent signaling of coincident auditory and visual events in primary visual cortex K. Svoboda, A. J. King, A. Mizrahi, & A. Holtmaat, eds. eLife 8:e44006.

Glickfeld LL, Olsen SR (2017) Higher-Order Areas of the Mouse Visual Cortex. Annu Rev Vis Sci 3:251–273.

Glickfeld LL, Reid RC, Andermann ML (2014) A mouse model of higher visual cortical function. Curr Opin Neurobiol 24:28–33.

Ibrahim LA, Mesik L, Ji X, Fang Q, Li H, Li Y, Zingg B, Zhang LI, Tao HW (2016) Cross-Modality Sharpening of Visual Cortical Processing through Layer-1-Mediated Inhibition and Disinhibition. Neuron 89:1031–1045.

Iurilli G, Ghezzi D, Olcese U, Lassi G, Nazzaro C, Tonini R, Tucci V, Benfenati F, Medini P (2012) Sound-Driven Synaptic Inhibition in Primary Visual Cortex. Neuron 73:814–828.

Matteucci G, Piasini E, Zoccolan D (2024) Unsupervised learning of mid-level visual representations. Curr Opin Neurobiol 84:102834.

Matteucci G, Zoccolan D (2020) Unsupervised experience with temporal continuity of the visual environment is causally involved in the development of V1 complex cells. Sci Adv 6:eaba3742.

Meijer GT, Montijn JS, Pennartz CMA, Lansink CS (2017) Audiovisual Modulation in Mouse Primary Visual Cortex Depends on Cross-Modal Stimulus Configuration and Congruency. J Neurosci 37:8783–8796.

Muratore P, Tafazoli S, Piasini E, Laio A, Zoccolan D (2022) Prune and distill: similar reformatting of image information along rat visual cortex and deep neural networks In Advances in Neural Information Processing Systems 34 .

Murray JD, Bernacchia A, Freedman DJ, Romo R, Wallis JD, Cai X, Padoa-Schioppa C, Pasternak T, Seo H, Lee D, Wang X-J (2014) A hierarchy of intrinsic timescales across primate cortex. Nat Neurosci 17:1661–1663.

Nayebi A, Kong NCL, Zhuang C, Gardner JL, Norcia AM, Yamins DLK (2023) Mouse visual cortex as a limited resource system that self-learns an ecologically-general representation. PLOS Comput Biol 19:e1011506.

Niell CM (2011) Exploring the Next Frontier of Mouse Vision. Neuron 72:889–892.

Oude Lohuis MN, Marchesi P, Olcese U, Pennartz CMA (2024) Triple dissociation of visual, auditory and motor processing in mouse primary visual cortex. Nat Neurosci:1–14.

Piasini E, Soltuzu L, Muratore P, Caramellino R, Vinken K, Op de Beeck H, Balasubramanian V, Zoccolan D (2021) Temporal stability of stimulus representation increases along rodent visual cortical hierarchies. Nat Commun 12:4448.

Tafazoli S, Safaai H, De Franceschi G, Rosselli FB, Vanzella W, Riggi M, Buffolo F, Panzeri S, Zoccolan D (2017) Emergence of transformation-tolerant representations of visual objects in rat lateral extrastriate cortex. eLife 6:e22794.
