# Supplementary material for: Signatures of hierarchical temporal processing in the mouse visual system
Source: PLoS Comput Biol. 2024 Aug 22;20(8):e1012355. doi: 10.1371/journal.pcbi.1012355 (PMC11373856; doi:10.1371/journal.pcbi.1012355)
Supplement: Attachment — Submitted filename: Letter_SignaturesTemporalHierarchy_PLOSCB_R1.pdf [file pcbi.1012355.s028.pdf]

MAX-PLANCK-GESELLSCHAFT

L. Rudelt & V. Priesemann

Max-Planck-Institute for Dynamics and Self-Organization
Am Faßberg 17
D-37077 Göttingen
✉ lucas.rudelt@ds.mpg.de

Göttingen, July 12, 2024

Resubmission of our manuscript to *PLOS Computational Biology*

Dear editors, dear reviewers,

we thank you for handling our manuscript "Signatures of hierarchical temporal processing in the mouse visual system". Please find enclosed a point-to-point response to the reviewer's comments, and a manuscript file with changes highlighted in color.

Most centrally, we rewrote and extended the discussion section and parts of the results for a more balanced account of the possible mechanisms underlying the hierarchy in timescales and predictability, and added key literature on the functional benefits of long timescales. We also renamed the intrinsic timescale to correlation timescale throughout the manuscript and clarified that this timescale captures both, stimulus-induced as well as intrinsic effects. Furthermore, we have corrected some parts in the introduction and discussion on hierarchical processing in rodents and primates.

In more detail, we have extended the discussion to better account for the possible role of increasingly invariant and complex sensory representations for the hierarchy of timescales and predictability. Whereas previously the discussion was written "either-or" and in favour of intrinsic mechanisms and adaptation, we now incorporate more literature on the fact that intrinsic mechanisms and feedforward sensory processing could be inextricably linked, and incorporated more literature on how hierarchical sensory representations could also lead to a decrease in predictability.

In this process, we have also renamed the intrinsic timescale to correlation timescale because the name intrinsic timescale should refer to a precise measure in the literature that only corresponds to the correlation timescale in our paper in the case of spontaneous activity. In contrast, when estimated for activity under visual stimulation, the correlation timescale reflects both stimulus-induced and intrinsic effects, hence the name intrinsic timescale is not that accurate in our case, as noted by one reviewer.

Finally, we have also re-implemented the analysis pipeline for better readability of the code and to facilitate reproduction.

We thank you for your editorial consideration and are looking forward to your reply.

Lucas Rudelt and Viola Priesemann on behalf of all authors

Reviewer 1

I liked this paper a lot, and I believe that the comparison of intrinsic vs coding time scales is of high importance. I have no concerns about the analyses and interpretations, which are done to a high standard. There are aspects about background literature and interpretations of results that would deserve a better elaboration and a little more thinking, to make sure the implications of the results are fully exploited and better placed in the context of the existing literature.

> Thank you very much for your time to review the paper. We are also thankful that you pointed us to this relevant literature. We addressed your comments point by point below.

The first relates to the relationship between timescales and efficient coding. There are a couple of papers that seems highly relevant and should be cited and discussed in this context. Młynarski and Hermundstad Nature Neuroscience 2021. Fairhall et al Nature 2001.

> This is an interesting thought that we did not consider yet. The fact that neural adaptation "memorizes" the timescale of slow stimulus variables is fascinating and seems an important aspect of temporal processing in sensory areas. We cite the two references in the methods, because we find an additional slow decay of the autocorrelation function that could be a result of slow stimulus variables. In particular, we think that the fact that the slowly decaying component of the autocorrelation is weaker than the faster decaying one could be a result of adaptation and efficient coding, which actively suppresses such slowly varying, highly predictable and thus redundant sources of stimulus information on a wide range of timescales. The relevant text in Lines 510ff. reads:
>
> *"Here, we defined the timescale with the larger coefficient A_i as the intrinsic (dominant) timescale $\tau_C = \tau_i$, whereas the other coefficient $A_{j\neq i}$ corresponds to a secondary timescale $\tau_{\mathrm{sec}} = \tau_j$ that accounted for complementary effects like slow stimulus variables. Note that for $\approx 80\%$ of units τ_{sec} was larger than τ_C, i.e., it mainly accounted for an additional, slow decay (see Supplementary Fig. S6 for a distribution of the fitted timescales). That slow stimulus variables have a smaller contribution to the autocorrelation is also in line with evidence for efficient coding, where neural representations were found to be strikingly invariant and adapt to slow stimulus variables through scale-free adaptation (Fairhall et al. 2001; Lundstrom et al. 2008; Młynarski and Hermundstad 2021)."*

The second is related to the temporal redundancy and its possible functional role. The discussion of this seems limited to the role that long timescales can have in predictability. An independent view of what could be the functional role of these timescales (which as the authors point out in the Introduction, can be due to coupling between cells not only to single-cell mechanisms), is that the benefits of long timescales for perceptual discriminations so far have been strongly imputed to the fact that these longer information timescales facilitate the downstream readout of information, relating to the fact that these timescales are due to or accompanied by stronger time-delayed coupling between cells. See Valente et al, Nature Neuroscience 2021. Panzeri et al Nature Reviews Neuroscience 2022. This evidence should be elaborated in the Discussion section about long information timescales.

> This is a great point, because our discussion on timescales was focused on the integration of past information for forming neural representations. However, ultimately, sensory information has to be represented in a way that is useful for a downstream readout. In Lines 395ff., we now discuss the relevance of long, stimulus independent information timescales and their link to stronger coupling between cells for downstream readout of information in the discussion part on neural coding, where we include the references that you proposed:

"In particular, our results indicate that intrinsic network mechanisms, such as an increase in recurrent coupling, are also important for shaping the temporal processing hierarchy. Such mechanisms are thought to cause stronger noise correlations in population codes (Panzeri et al. 2022), which were found to yield long timescales of information consistency and more stable representations for better readout and evidence accumulation in higher cortical areas (Morcos and Harvey 2016; Piasini et al. 2021; Valente et al. 2021)."

Reviewer 2

In this manuscript, the authors quantified the timescale of neural activity in mouse visual cortex using two metrics: decaying time of single-neuron autocorrelation function and an information-theoretic metric, the predictability, which yields both a timescale and an asymptotic value. They found a clear increase in both timescales when moving from thalamic regions to primary visual cortex and further up along the higher cortical areas. The total predictability rose from thalamus to visual cortex, but decreased along the visual hierarchy (as measured by an anatomical score). Importantly, Bayesian modeling showed that the within-visual-cortex timescale trends were robust and significant and a two-stage (V1 - higher areas) model did a worse job at reproducing the data then a (more-complex) fully hierarchical model. This was not true for overall predictability. In addition, the authors found a very similar hierarchical increase of time scales when analyzing spontaneous activity (in absence of visual stimuli). Finally, the authors introduce a conceptual model to show how the co-existence of sufficient recurrent activity amplification (a network effect) and single-neuron predictability source can qualitatively reproduce the hierarchical increase in timescales and decrease in overall predictability. Overall, I found the paper very interesting, with some innovative and imaginative analyses (like the mutual info predictability measure) that, I believe, will turn out to be very useful in the field. Another innovative element of the study is the comparison of time scales with and without visual stimulation. The fact that similar trends were obtained in the two conditions is far from obvious and, as the authors concluded, does suggest a dominant effect of intrinsic, recurrent processing, at least under some conditions.

At the same time, I found one major issue in the study, which is the mismatch between what the authors claimed to have computed (i.e., INTRINSIC timescales) and what they have actually computed, which are metrics that depend on both intrinsic and stimulus-driven processes, as far as I can understand. This semantic mismatch does requires, in my opinion, a revision of some of the statements and conclusions of the manuscript. In addition, I believe that a more direct comparison with similar work performed by previous studies would help putting the results of the authors in a broader context and clarify some of their interpretation. Full disclosure here: I am Davide Zoccolan, the author of a study (Piasini et al 2021) that addressed very similar questions to those addressed by the authors. I prefer to disclose my name, since many of the criticisms and suggestions I have listed below derive from my very direct experience in dealing with similar data and questions. So, I believe that all my comments will be more understandable if the authors know where they come from.

> Thank you very much for your time to review this paper, for all your helpful comments and the careful assessment of our manuscript. We addressed your comments point-by-point below.

MAJOR POINTS

1) As I mentioned above, the major issue I encountered in reading the manuscript is the way the authors defined the intrinsic timescales. This issue is strictly related to the way the authors formatted the Allen data. So, I refer here to the Methods sections named "Experimental data sets" and "Estimation of intrinsic timescale".

The first aspect of the methods that is not fully clear to me is how the authors handled the neuronal responses collected across repeated presentations of the same movie. Take for instance natural_movie_one_more_repeats. As the authors wrote, this portion of the dataset contains 15 min of neuronal responses to the repeated presentation of a 30 second naturalistic movie. How are the authors dealing with such repeated presentations? Are they simply ignoring them, treating, de facto, the 15 min of recording as a unique trial of a single movie lasting many minutes (even if, in fact, the movie is made of many repeated, identical sub-movies)? Or do the authors instead extract each single presentation of the 30 sec clip to create a dataset where they look at the trial-by-trial variability of the neuronal response to this 30 sec clip, as well as compute the trial-average firing rate during the presentation of the 30 sec clip? At some point, the authors write "natural movie blocks were concatenated to then yield a total recording length of 18 minutes." Based on this sentence, it would seem to me that the authors processed

the data in the first way, i.e., as a unique, 18 min long movie (not considering the periodic nature of the actual, repeated stimulus).

This is important to understand, because, if so, this means that all the analyses of the study are performed at the single trial level (ignoring the statistical variability of the response across repeated trials). This seems consistent with their definition of the autocorrelation function from which they extracted the intrinsic time scale. In eq. 1, they simple refer to the binary activations a_t of the neurons, without mentioning whether they are computed across repeated presentations/trials of the movie clip. The average seems to be computed across time only and not across trials. This way to compute autocorrelation is fully legitimate of course, but it should be well understood what it implies in terms of the underlying neuronal process. For instance, the authors refer to the resulting time scale as "INTRINSIC", but this is not what most authors have referred to as "intrinsic time scale" in previous studies. Intrinsic time scale is the one that is computed by measuring the correlation coefficient between the responses fired across different trials/repetitions of the same stimulus in different time bins during the recording. This is well defined, for instance, in (Murray et al., 2014), which calls it "the across-trial correlation between spike counts". As explained by Murray, this autocorrelation "corrects for nonstationarity in the mean firing rate during the foreperiod (for example, ramping) because covariance and variance subtract the mean spike count for each time bin". This means that this way of computing the autocorrelation is basically independent from the actual magnitude of the firing rate - e.g., it is independent from the stimulus-driven component of the response. It does only depend on the stochastic fluctuations of the response around its mean across different trials. In this sense it is "intrinsic" - it measures how likely it is that a cell, having fired, at a given time, during a given trial, above its across-trials average, will keep doing so, at following times during the same trial. It is a measure of "inertia" of the firing rate, which should be largely independent from the underlying stimulus producing the response. It is equivalent to the noise correlation typically computed between pairs of neurons – only, this is a noise autocorrelation computed for the same neuron across different times.

We explained even more explicitly the different nature of intrinsic and stimulus-driven (or response) time scales in (Piasini et al., 2021), where we have used the same approach of Murray to compute the intrinsic time scales, but we have also computed the stimulus driven time scale by measuring the autocorrelation function of the trial-averaged firing rate (i.e., of the PSTH of the response). In the latter, being computed over a trial-averaged response, the intrinsic dynamic of trial-by-trial fluctuations is lost. This yields a pure measure of the stimulus-induced autocorrelation of the response over time.

Now, if I have well understood what the authors did, their autocorrelation is neither purely intrinsic, nor purely stimulus driven. It is a mixture of the two. Being computed on a single trial, it does reflect the stability of the response in encoding the stimulus. At the same time, it also reflects the intrinsic noise auto-correlation. Again, nothing wrong with this, provided that the authors properly explain what their autocorrelation means and they name it properly. In fact, the resulting time scale cannot possibly be called "intrinsic". That would be quite misleading and incorrect with respected to the definitions used in the previous literature and the meaning itself of what is measured. As such, I believe that the authors should revise all instances where they refer to this measure as "intrinsic time scale" and rename it. Not sure what a good name can be, perhaps "within-trial, response timescale"? I want to point out that all these arguments apply also to the information time scale the authors extract from their predictability measure. Since, also in this case, predictability is computed from a minutes-long single trial (ignoring the trial-by- trial variability of the response), the resulting metric (and its time scale) depend both on the component of the response that is stimulus-determined and by the noise autocorrelation (i.e., the intrinsic dynamic) of the response. I believe the authors should be well aware of this, when they interpret their findings on the hierarchy of time scales.

> This is a very important point that you are addressing in order to prevent any misunderstanding and misinterpretation of the results. You are right about the way we computed timescales and predictability, they are based on ongoing activity, taking repeated movies

as one stream of visual input and assessing the statistics of neural activity under this continuous visual input. We clarify this now at the beginning of the results section where we introduce the timescale and predictability measures (see below).

In the case of natural movies, the timescales and predictability thus capture both, intrinsic and stimulus-induced correlations. For the autocorrelation, we had called the measure intrinsic timescale, because, as you pointed out, it is analogous to the intrinsic timescale from previous studies (e.g., Murray et al. 2014) in the case of spontaneous activity. However, we agree that it is misleading to refer to it as intrinsic timescale throughout the paper, because it clearly does not correspond to the intrinsic timescale in the natural movie condition. Therefore, we renamed the measure, referring to it as "correlation timescale" because it is based on autocorrelation in contrast to the information timescale, which is based on mutual information.

We clarified that the estimated timescales and predictability reflect both stimulus-induced and intrinsic effects at the beginning of the results section, where we also explain how the correlation timescale is related to the intrinsic timescale. The corresponding part in Lines 116ff. reads:

"Using the autocorrelation, we estimate the correlation timescale of spiking activity, which is computed as the decay time τ_C of an exponentially decaying autocorrelation (Fig. 1A, Methods). It is important to note that the resulting correlation timescale captures temporal correlations from both, intrinsic neural mechanisms and visual stimuli. One way to disentangle these contributions is to work with trial-based data where mean non-stationary inputs can be estimated and removed (Murray et al. 2014; Heuvel et al. 2020; Piasini et al. 2021), which gives rise to the so-called intrinsic timescale (Murray et al. 2014). Here, we instead compute the correlation timescale for ongoing activity in an entire stimulus block to obtain clean estimates on the single-neuron level. We then systematically compare the estimated timescale under stimulation with a natural movie with the one obtained for spontaneous activity, where the latter reflects the intrinsic timescale (Jens Wilting and Viola Priesemann 2018)."

and in Lines 129–131

"Similar to the correlation timescale, the predictability and information timescale reflect both stimulus-induced and intrinsic effects, and thus will be systematically compared between stimulus conditions."

Finally, we have slightly rewritten the abstract to be more clear in this respect. Lines 14–18 now read:

"Here, we used an optimized information-theoretic and classical autocorrelation analysis to show that information- and correlation timescales of spiking activity increase along the anatomical hierarchy of the mouse visual system under visual stimulation, while information-theoretic predictability decreases. Moreover, intrinsic timescales for spontaneous activity displayed a similar hierarchy, whereas the hierarchy of predictability was stimulus-dependent."

2) Lines 160-162 "In contrast, median predictability decreased (rP = −0.93), which might reflect an increase of adaptation that attenuates responses to predictable stimuli for an efficient code". Also at lines 227-229: "This also suggests that the decrease in predictability along the hierarchy might reflect an active cancellation of predictability in visual stimuli, e.g., through stronger adaptation in these areas."

The result about the hierarchical increase of information time scales and decrease of predictability is very intriguing. Here and in the Discussion, the authors propose an interpretation of this finding in terms of adaptation, and/or predictive/efficient coding. This is certainly a valid hypothesis, but other (simpler) hypotheses should also be taken into account (perhaps in the Discussion).

In particular, I believe that the information theoretic analysis performed by the authors tap onto the same mechanisms/properties we have explored in (Piasini et al., 2021) with a linear decoding analysis (see Fig. 6 of our study). The two approaches are not identical of course, but I think they basically show the same phenomenon. In their analysis, the authors measure how well past activity can predict the current one. They see that the deeper they along the hierarchy, the longer is the dependency from past activity, but the less is the info provided by past activity in terms of magnitude. In our analysis, we trained a linear decoder at time t_0 and then we tested how well that decoder was able to discriminate movie segments at following times. We found that, in terms of magnitude, the accuracy was larger in lower visual areas, but in terms of stability of the accuracy, this was much larger in deeper areas. This seems to be consistent (if no equivalent) to what found by the authors with their analysis. Early neuronal representations seem to contain more information about the visual input and this information, over short time scales, remain stable enough to allow, in our case, good generalization of decoding accuracy, and, in their case, good predictability. Higher areas contain less stimulus info, but, whatever they are able to represent, it remains way more stable over time.

Why this happens? The interpretation we gave of this finding in (Piasini et al., 2021) is that this is due to the much higher sensitivity of neurons in early visual areas to low-level visual features (like contrast, luminance, position, etc.), as compared to higher-order neurons. We have shown this in a previous study based on info theory (Tafazoli et al., 2017). The hypothesis we put forward in (Piasini et al., 2021) was that these low-level features remain quite informative about the stimulus (e.g., object identity) over short time scales, but they rapidly become poorly informative over long time scales. By contrast, in deep areas, most of the low-level info has been pruned - see again (Tafazoli et al., 2017), but also (Muratore et al., 2022). So, these representations are less able to discriminate the visual input (in terms of magnitude of the decoding accuracy or of the mutual info between stimulus and response) and, at short time lags, the discrimination accuracy they afford is going to be lower than in lower areas. But these higher areas encode more invariantly higher-order features and, because of this, they are able to maintain their discriminatory power very stable over time.

So, I believe that the analysis of the authors is quite consistent with this finding and interpretation of our previous study. And I believe it would be worth mentioning it as an alternative explanation of the concomitant increase of information time scale and decrease of predictability magnitude.

> Thank you for pointing out this additional, intriguing hypothesis about the decay of predictability along the processing hierarchy. We agree with your reasoning that simple-cell responses could be overall more predictable due to the stronger modulation by predictable visual input, whereas complex cells are tuned to features that are correlated over longer times, but overall less predictable.
>
> Therefore, we extended the discussion in Lines 419ff. to include this interpretation:
>
> *"Finally, a decrease in predictability could be the result of complex, temporally invariant input representations formed in higher areas. These invariant representations have been shown to contain less stimulus information in absolute terms, because most of the information about low-level features such as contrast, luminance, position or phase is pruned in higher areas (Tafazoli et al. 2017; Muratore et al. 2022), which also results in worse stimulus decoding performance for these areas (Piasini et al. 2021). Our results on predictability are compatible with this idea, because predictability is positively correlated with stimulus selectivity and higher under visual stimulation (Fig. 3), hence a decrease in predictability can be related to a decrease of absolute stimulus information for higher areas. Notably, all mechanisms may co-exist to serve complementary functions: Recurrence could enable long integration for temporal processing on the network level, while adaptation could ensure efficiency of the neural code on a single-neuron level, and both mechanisms could aid the formation of invariant, high-level representations by supporting temporal learning and the pruning of low-level, highly predictable information in higher cortical areas."*

We also changed the following two passages in Lines 170-172 and Lines 242-246 to be either more neutral, or also incorporating this additional interpretation:

"In contrast, median predictability decreased ($r_P = -0.92$), which indicates less temporal redundancy within individual spike trains in higher cortical areas."

and

"Together, these results imply that the hierarchy of timescales is supported by network-intrinsic mechanisms (independent of stimulus conditions or visual coding properties), whereas the hierarchy of predictability is more closely related to visual input. This also suggests that the decrease in predictability along the hierarchy might reflect an active cancellation of predictability in visual stimuli, or is an inherent property of higher-level visual representations (see Discussion)."

3) Lines 225-226. "Together, these results imply that the hierarchy of timescales is supported by network-intrinsic mechanisms (independent of stimulus conditions or visual coding properties)".

The result on the spontaneous activity is very interesting and, to some extent, surprising. Especially the fact that the hierarchy of temporal scales is also present in that condition. But I believe that the interpretation of this finding is more nuanced and complex than what stated here and in the Discussion. As I mentioned, the authors did not compute intrinsic time scales, but the time scale of the response resulting from a combination of intrinsic dynamics (noise autocorrelations) and stimulus induced dynamics. In (Piasini et al., 2021), we have shown that, in the Allen data, both time scales (when measured separately) increase hierarchically. In the test of the authors with spontaneous activity, there is no dynamic stimulus, so, although their metric is not, in general, a pure measure of intrinsic dynamics, we can safely assume that, under absence of stimulation, it mainly reflects intrinsic dynamics. That is, the increase of time scales across areas must be due, in this case, purely to noise autocorrelations. In such a condition, I would expect to still see the hierarchical trend, but perhaps weaker as compared to the case where a dynamic stimulus is present (because one of the two component that sets the time scale of the dynamics is gone - the stimulus-driven one). I guess I can see a hint of this by comparing the trend of Fig. 2 and Supp Fig. 12. In Fig. 2D, for instance, the range of time scales spanned by the areas is between 250 and 450 ms, while in Fig. S12D is from 300 to about 400 ms. It would be interesting if the authors could statistically test this question: is the hierarchal increase becoming less sharp in the spontaneous activity? Can the authors apply their Bayesian analysis to explicitly test this? Just as a reference: in our study we did observe, for the rat dataset, that intrinsic dynamics did add further stability to the one already afforded by the progressively invariant encoding of shape information - see Fig. 6D of (Piasini et al., 2021).

Thank you for pointing this out. We think that it is a very interesting perspective that intrinsic mechanisms complement stimulus coding through feedforward mechanisms, as opposed to an "either-or" solution. We have therefore rewritten the part in Lines 225-226 to state that our results imply that network intrinsic effects *support* the hierarchy in timescales (see reply to previous point).

It is also an interesting question whether we see that the stimulus encoding "adds" to this hierarchy, i.e., sharpens the effect. It is hard to explicitly test this. However, we have plotted the posterior distributions of the slope parameters in the Bayesian analysis in Supplementary Fig. 13 (for convenience shown below), where one can see that for the correlation timescale the posterior distributions for the mean hierarchy slope are very similar between all three stimulus conditions. However, for the information timescale, the posterior for the mean hierarchy slope clearly indicates a smaller slope for spontaneous activity when compared to the two natural movie conditions (Fig. 13H, posterior median lies below the 95% highest density intervals for both natural movie conditions). We also observe a smaller linear correlation with the hierarchy score for the information timescale in Supplementary

Fig 12. This suggests that the stimulus representation could indeed add to the observed hierarchy in information timescales. We do now mention this result in Lines 212ff. of Results:

"When comparing timescales between the previous natural movie condition and spontaneous activity that was recorded while showing the animal a grey screen (Methods), we found that median τ_C and τ_R were similar between stimulus conditions (pooled from all units in all cortical areas, Fig. 3A,B). Moreover, we found that a cortical hierarchy of timescales also exists for spontaneous activity (Supplementary Figs. 12, 13 and 15). This hierarchy was strikingly similar for the correlation timescale, whereas for the information timescale the correlation with the hierarchy score was smaller under spontaneous activity (Supplementary Fig. 12) and the Bayesian regression indicated a smaller mean slope (Supplementary Fig. 13). This suggests that the hierarchy of correlation timescales is little affected by the stimulus condition, while the hierarchy of information timescales becomes more pronounced under visual stimulation."

Finally, we have revised the Coding perspective part in the discussion to include this result, where we also refer to the result of Piasini et al. 2021 that intrinsic dynamics add further stability to the one already afforded by the invariant encoding (see reply to next point).

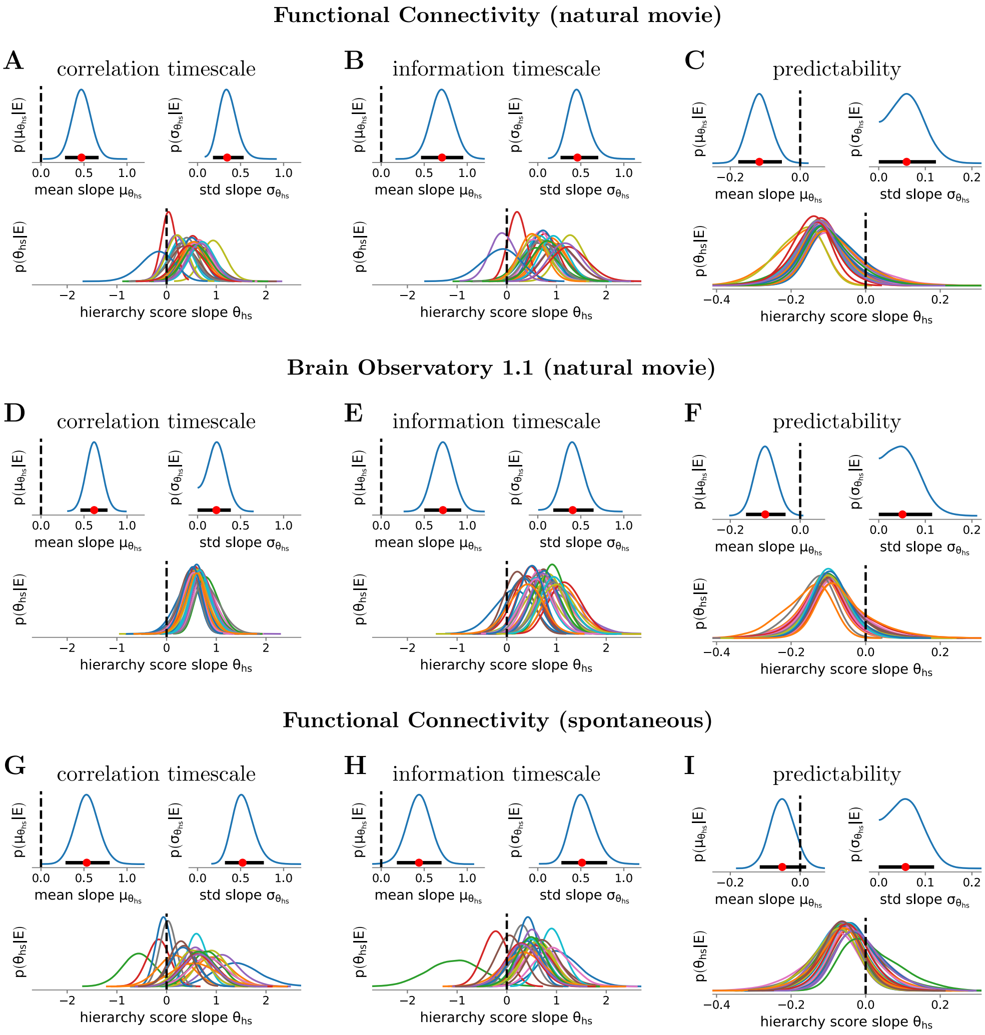

Figure S13. Posterior distributions of the hierarchy score slope reveal a significant increase in timescales and decrease in predictability with hierarchy score. To assess whether

timescales and predictability relate to the anatomical cortical hierarchy, a linear relationship between an area's median and anatomical hierarchy score was modelled with slope θ_{hs}. **(A)** (Top) For the correlation timescale, the 95% posterior credible interval of the mean hierarchy score slope $\mu_{\theta_{\mathrm{hs}}}$ across all mice is positive (black bar, red dot indicates median), indicating that there is an increase in median correlation timescales with the hierarchy score. (Bottom) On the level of individual mice, posteriors indicate the same effect, but are more diverse (colors indicate different mice). In particular, for some mice the posteriors also attribute probability to zero or negative slopes, which could be either due to increased uncertainty due to the smaller sampling size, or an incomplete sampling of the areas for individual mice. **(B)** For the information timescale τ_R, the posterior credible interval of the mean slope is also positive. **(C)** For predictability, in contrast, the credible interval is negative, indicating a credible decrease in predictability with hierarchy score. **(D–F)** Very similar results are obtained for the *Brain Observatory* data set. **(G)** For spontaneous activity and the correlation timescale, the posterior of the mean slope is very similar to the natural movie conditions, but variability across mice is larger. **(H)** For the information timescale, in contrast, the posterior indicates a smaller slope. **(I)** For predictability, the credible interval even contains a zero slope, indicating that predictability does not necessarily decrease along the anatomical hierarchy for spontaneous activity.

4) Discussion section. In general, I believe that the authors should revise their Discussion to consider more alternative hypotheses (see my comments above), and to make sure they do not misinterpret or overinterpret their findings, in the light of the fact that the timescales they have measured are not purely due to intrinsic processes. More specifically, I report below a few sections that, I believe, require the attention of the authors.

Lines 286-289: "We concluded that the gradients in timescales are therefore likely caused by intrinsic mechanisms, like recurrent coupling that could serve an enhanced temporal integration for higher areas [1, 15, 25, 26, 41], whereas a reduction of predictability might be the result of stronger cancellation of redundant, predictable stimuli, in line with hierarchical predictive and efficient coding". I believe that this statement is too strong, in the light of the fact that what the authors measured is not about pure intrinsic dynamics and in the light of our findings with both rats and mice, where we did find a hierarchal increase of timescales also for the trial-averaged responses, where there is no contribution from intrinsic dynamics (Piasini et al., 2021).

We agree with your concern and have revised this part in lines 301ff., which now reads:

"Moreover, we found similar gradients and median values of timescales under spontaneous activity, indicating that the observed gradients in timescales are also shaped by network intrinsic mechanisms like an increase in recurrent coupling that could serve an enhanced temporal integration for higher areas (Jens Wilting, Dehning, et al. 2018; J. Wilting and V. Priesemann 2019; Chaudhuri et al. 2015; Zeraati et al. 2023; Runyan et al. 2017). In contrast, we observed a decrease in predictability mainly under stimulation with a natural movie, which could be the result of an overall higher sensitivity of lower areas to lower-level sensory features with higher predictability (Tafazoli et al. 2017; Muratore et al. 2022), or a stronger cancellation of redundant, predictable stimuli, in line with hierarchical predictive and efficient coding (Rao and Ballard 1999; Simoncelli 2003)."

Lines 381-387: "... Here, we found that timescales hardly change with visual stimulation and stimulus selectivity of the units (Fig. 3), ...This is also consistent with findings from rat visual cortex [40], ..., these results suggest that long intrinsic and information timescales rely primarily on properties of the network, e.g., recurrence, rather than temporal properties of the inputs".

Again, the authors need to be careful here with this interpretation. Their arguments rest on the assumption that they have measured pure intrinsic time scales, which is not the case. In addition, the authors seem to conclude that the timescale they measured are stimulus-independent, so largely to due to recurrent connectivity, because of their findings with spontaneous activity and because they "timescales hardly change with stimulus selectivity of the units". However, the latter argument is weak in my opinion, because the most important tuning property that should have been assessed in order to support this claim is whether the recorded cells were more simple-like (i.e., phase sensitive) or more complex-like (i.e., more phase invariant). The former would be expected to show faster time scales than the latter, because of the larger translation/phase-invariance of the latter. We did find this distinction in V1 [see (Matteucci and Zoccolan, 2020), Fig. 4 and 5]. Since the authors have not measured this property, it is unclear what to expect from their analysis - I mean, there is no clear prediction, as far as I can think, about whether timescales should depend in some way or another from direction selectivity.

> We agree that also here the discussion of the results was not balanced. We have revised this discussion paragraph to state that intrinsic mechanisms do play a role, instead of being the primary cause for long correlation and information timescales. We have also incorporated the result that visual stimulation seems to "sharpen" the hierarchy in information timescales, and cite your result that vice versa intrinsic mechanisms enhance the stability of the invariant encoding formed by higher areas. Finally, we find the idea fascinating that intrinsic mechanisms and invariant stimulus representations could be related through unsupervised temporal learning (Matteucci and Zoccolan 2024), which we also incorporated into the discussion. All in all, this discussion part in Lines 387ff. now reads:
>
> *"Turning to a coding perspective, a hierarchy in timescales could also result from a feedforward representation of increasingly more complex visual features: A successive integration of temporally short-lived, simple features into temporally long-lived, complex ones could lead to long timescales in those neurons that represent the complex features (Hasson, Yang, et al. 2008; Hasson, Chen, et al. 2015; DiCarlo, Zoccolan, et al. 2012; Piasini et al. 2021), which was found empirically in rat V1 (Matteucci and Zoccolan 2020). In line with this, we found the hierarchy in information timescales to be more shallow under spontaneous activity (Supplementary Figs. 12 and 13), indicating that visual stimulation has an effect on the information timescale hierarchy. However, we also found that median timescales and the correlation timescale hierarchy were very similar between visual stimulation and spontaneous activity, (Fig. 3 and Supplementary Figs. 12 and 13), mirroring findings in monkey somatosensory cortex (Rossi-Pool et al. 2021). This indicates that stimulus encoding and complex feature representations do play a role, but cannot be the sole cause for long correlation and information timescales.*
>
> *In particular, our results indicate that network-intrinsic mechanisms, such as an increase in recurrent coupling, are also important for shaping the temporal processing hierarchy. Such mechanisms are thought to cause stronger noise correlations in population codes (Panzeri et al. 2022), which were found to yield long timescales of information consistency and more stable representations for better readout and evidence accumulation in higher cortical areas (Morcos and Harvey 2016; Piasini et al. 2021; Valente et al. 2021).*
>
> *However, feedforward feature representation and long timescales through intrinsic mechanisms are not necessarily separate processes. There exists the exciting possibility that the intrinsic generation of long timescales in neural activity could contribute to the activity-dependent learning of temporally invariant complex visual features (Matteucci and Zoccolan 2020; Matteucci, Piasini, et al. 2024). In this case, feedforward and recurrent processing would be inextricably linked for temporal processing, which could explain the similarity in median timescales across stimulus conditions."*

Lines 388-389: "Of particular interest from a coding perspective are our results on predictability Rtot, because they imply that the temporal redundancy in spike trains decreases along the hierarchy". And

also lines 414-416: "In contrast, the stimulus-dependent decrease in predictability is in agreement with efficient coding as well as an observed increase of adaptation in rodents, and might constitute another hallmark of hierarchical temporal processing". These statements are too strong. I believe that here it should be discussed the alternative hypothesis I mentioned at point 2. That is, it could be the higher sensitivity of early visual areas to low level features (i.e., the richer, more faithful representation of visual images) to allow Rtot to be larger in these areas and then to gradually decrease in higher order areas. This would be a purely feedforward mechanism that does not involve increased adaptation or predictive signals across the hierarchy.

> We agree that the discussion has been too one-sided, and have added a paragraph in the discussion in Lines 419ff. that elaborates on the alternative hypothesis that you have provided here, and points out that the different mechanisms might also complement each other (see reply to Point 2). Similarly, we have revised the conclusion paragraph in Lines 442ff. that you have been referring to, which now reads:
>
> *"Our analysis of data from different stimulus conditions and recurrent network models further indicated that the gradients in timescales are supported by stimulus-independent, network-intrinsic mechanisms, such as an increasing recurrence along the anatomical hierarchy. In contrast, the stimulus-dependent decrease in predictability is in agreement with efficient coding and the observed increase of adaptation in rodents, as well as a formation of increasingly invariant, high-level visual representations, and might constitute another hallmark of hierarchical temporal processing in mammals."*
>
> Finally, regarding the sentence in Lines 388-389 we argue that this statement is independent of the underlying mechanism and only refers to the fact that predictability is an information theoretic measure of redundancy. Therefore, we think that this statement is justified and we would like to leave it as it is.

MINOR POINTS

1) Lines 61-63. "there seems to be no clear global processing hierarchy from sensory to transmodal areas [29], as more of mouse cortex is used for sensory processing of specific modalities, instead of integrating information across modalities".

I only partially agree with the first part of the sentence: functional evidence of hierarchical processing is indeed weak in the mouse. E.g., see some recent modeling studies of neuronal representations of natural images along the mouse visual system, where the hierarchy seems quite shallow (Cadena et al., 2019; Nayebi et al., 2023). At the same time, some other studies (like the Allen paper itself from which the authors took the data) suggest the existence of a quite clear hierarchy in terms of a number of basic tuning and response properties. In addition, at the anatomical level, the evidence of a hierarchy is quite substantial, and suggests even the presence of a ventral and a dorsal pathway. In addition to the Allen studies, I suggest looking at the work of Burkhalter's lab or to a few reviews on the topic (Niell, 2011; Glickfeld et al., 2014; Glickfeld and Olsen, 2017). So, the authors should be a bit more nuanced in their statement here and refer to a wider pool of studies to support their statement.

Also, I disagree with the second part of the sentence: "as more of mouse cortex is used for sensory processing of specific modalities, instead of integrating information across modalities". If anything, sensory cortices in rodents are more multimodal than in primates. E.g., V1, A1 and S1 are coupled by horizontal cortico-cortical projections that are not quite present in monkeys. Se for instance: (Iurilli et al., 2012; Ibrahim et al., 2016; Meijer et al., 2017; Deneux et al., 2019; Oude Lohuis et al., 2024). It is true that we do not quite understand the role played by this cross-modal inputs to primary sensory cortices, but for sure it is not correct to state that mouse cortex is very unimodal.

We agree with your points. The intention here was to express that the hierarchy in mouse visual cortex appears to be more shallow, with overall fewer higher areas that integrate inputs from lower areas of different modality. Instead, transmodal integration seems to happen at a relatively early stage in processing through direct links between areas that are still specialized for a particular modality. Due to a very unfortunate choice of wording this part ended up conveying the exact opposite.

Regarding the first part of the sentence, we also agree that the statement is only partially correct. In fact, we realized that this paragraph and the subsequent paragraph did not convey the points that we wanted to make because they were not put well into context and lacked clarity. Thanks to your suggested references we were able to carve out a much clearer paragraph with statements that are better supported by literature. We also changed the order of the two paragraphs. The two paragraphs in Lines 61ff. now read:

"Here, we investigate mouse visual cortex because it comprises an anatomical and functional sensory processing hierarchy that differs from primate visual cortex in many interesting ways (Glickfeld and Olsen 2017). Visual cortex in mice exhibits clear hierarchical feedforward–feedback projection patterns (Harris et al. 2019), which are paralleled by differences in the recruitment of inhibitory and excitatory neurons (D'Souza et al. 2016), and a functional hierarchy that follows the anatomical hierarchy (Siegle et al. 2021). In addition, the rodent analog of the ventral stream shows a hierarchy of temporal scales where higher areas encode visual information more persistently (Piasini et al. 2021). Finally, population codes vary between association and sensory cortices (Runyan et al. 2017), and impairments to cortical frontal (transmodal) areas have a greater impact on evidence accumulation over long timescales than posterior (sensory) areas, which also exhibit shorter activity timescales during evidence accumulation (Pinto et al. 2022). Thus, the mouse visual cortex also shows many signatures of hierarchical temporal processing.

However, the mouse visual cortex was found to differ substantially from primate cortical organization. First, the sensory processing hierarchy seems to be more shallow (Huntenburg et al. 2021; Cadena et al. 2019; Nayebi et al. 2023), with overall fewer and more primitive higher areas (Glickfeld, Reid, et al. 2014), and multisensory integration at a relatively early stage in processing compared to primates (Mao et al. 2011). Second, although there exists evidence for gradients in interneuron numbers and intra-cortical connectivity from sensory to transmodal areas (Fulcher et al. 2019), the degree of interareal variation of microstructural properties in mice (Ballesteros-Yáñez et al. 2006; Benavides-Piccione et al. 2006) is far less pronounced than in the highly differentiated primate cortex (Elston and Fujita 2014; Gilman et al. 2017; Hsu et al. 2017; Luebke 2017). This raises the question whether a temporal hierarchy shaped by recurrence also characterizes mice, or whether the strong focus on sensory processing in a more shallow hierarchy requires a different, coding-optimized organization altogether."

2) Lines 192-196. "For rodents, a prominent hypothesis is that feedforward processing leads to more persistent stimulus representations for higher areas [40], thereby causing the longer timescales under visual stimulation. An alternative hypothesis is that stronger recurrence enables longer integration of visual information, similar to what is thought to cause longer timescales in primates".

The way in which this paragraph is written is a bit misleading. The authors seem to claim that the "feedforward" hypothesis is specific to the rodent visual system, while the "recurrent" hypothesis stems from the primate literature. However, at least insofar the "feedforward" hypothesis is concerned, the opposite is true – this hypothesis stems from what it is known about the monkey ventral stream at both the empirical and theoretical level. Empirically, the increase of invariance to transformations of static images should result in an increased temporal stability of the representations, when dynamic stimuli are used. From a computational point of view, learning of transformation- invariant representations is thought to be mediated by unsupervised adaptation to the temporal continuity of the visual input (the

so-called unsupervised temporal learning), thus resulting, again, in progressively slower representations along the ventral stream – see (Matteucci et al., 2024) for a review. The problem is that this hypothesis has never been quite systematically tested along the monkey visual stream. The fact that, in (Piasini et al., 2021), we managed to test it in the rat homologue of the ventral stream, does not mean, however, that the hypothesis was actually born in the rodent literature. So, the way in which this hypothesis is presented here should be corrected, giving the proper credit to the monkey ventral stream literature and the computational models inspired by this literature.

> This is a very good point. We incorporated additional references to give credit to the theoretical and empirical work on the monkey ventral stream and changed this part so that the first hypothesis is not exclusively associated with the rodent literature. The revised part in Lines 203–206 now reads:
>
> *"One hypothesis is that feedforward processing leads to longer timescales under visual stimulation because of transformation invariant, temporally persistent stimulus representations in higher areas (Riesenhuber and Poggio 1999; DiCarlo and Cox 2007; DiCarlo, Zoccolan, et al. 2012; Piasini et al. 2021). An alternative hypothesis is that stronger recurrence enables longer integration of visual information (Jens Wilting, Dehning, et al. 2018), which is thought to be a cause of long timescales in primates (Chaudhuri et al. 2015)."*

3) Lines 328-330: "whereas latero- and posteromedial areas LM and PM are thought to play a stronger role in object perception, analogous to the dorsal and ventral streams described in other species". Here, attention should be paid not to confound putative ventral and dorsal stream areas of the mouse cortex. LM, along with LI, P and POR are thought to be part of the mouse ventral stream. Instead, PM, along with AM, A, AL and RL are thought to be part of the dorsal stream – see (Niell, 2011; Glickfeld et al., 2014; Glickfeld and Olsen, 2017) and the work (already cited by the authors) of Burkhalter's lab.

> You are absolutely right; we mistakenly confused area PM with area P, incorrectly attributing it to the ventral stream. We have corrected this error, and the revised paragraph in Lines 342ff. now reads as follows:
>
> *"However, we also found deviations from a single gradient in timescales. In particular, the rostro-lateral area (RL) consistently had the smallest median timescales among extrastriate areas that were more comparable to primary visual cortex, despite its higher anatomical hierarchy score compared to the lateromedial area (LM). This might be due to the functional specialization of the posterior parietal areas (RL, AL, AM) and the posteromedial area (PM), which are thought to process information related to motion and behavioral actions, whereas LM is thought to play a stronger role in object perception, analogous to the dorsal and ventral streams described in other species (Niell 2011; Wang, Gao, et al. 2011; Wang, Sporns, et al. 2012)."*

REFERENCES

Cadena SA, Sinz FH, Muhammad T, Froudarakis E, Cobos E, Walker EY, Reimer J, Bethge M, Tolias A, Ecker AS (2019) How well do deep neural networks trained on object recognition characterize the mouse visual system? In NeurIPS Neuro AI Workshop .

Deneux T, Harrell ER, Kempf A, Ceballo S, Filipchuk A, Bathellier B (2019) Context-dependent signaling of coincident auditory and visual events in primary visual cortex K. Svoboda, A. J. King, A. Mizrahi, & A. Holtmaat, eds. eLife 8:e44006.

Glickfeld LL, Olsen SR (2017) Higher-Order Areas of the Mouse Visual Cortex. Annu Rev Vis Sci 3:251–273.

Glickfeld LL, Reid RC, Andermann ML (2014) A mouse model of higher visual cortical function. Curr Opin Neurobiol 24:28–33.

Ibrahim LA, Mesik L, Ji X, Fang Q, Li H, Li Y, Zingg B, Zhang LI, Tao HW (2016) Cross-Modality Sharpening of Visual Cortical Processing through Layer-1-Mediated Inhibition and Disinhibition. Neuron 89:1031–1045.

Iurilli G, Ghezzi D, Olcese U, Lassi G, Nazzaro C, Tonini R, Tucci V, Benfenati F, Medini P (2012) Sound-Driven Synaptic Inhibition in Primary Visual Cortex. Neuron 73:814–828.

Matteucci G, Piasini E, Zoccolan D (2024) Unsupervised learning of mid-level visual representations. Curr Opin Neurobiol 84:102834.

Matteucci G, Zoccolan D (2020) Unsupervised experience with temporal continuity of the visual environment is causally involved in the development of V1 complex cells. Sci Adv 6:eaba3742.

Meijer GT, Montijn JS, Pennartz CMA, Lansink CS (2017) Audiovisual Modulation in Mouse Primary Visual Cortex Depends on Cross-Modal Stimulus Configuration and Congruency. J Neurosci 37:8783–8796.

Muratore P, Tafazoli S, Piasini E, Laio A, Zoccolan D (2022) Prune and distill: similar reformatting of image information along rat visual cortex and deep neural networks In Advances in Neural Information Processing Systems 34 .

Murray JD, Bernacchia A, Freedman DJ, Romo R, Wallis JD, Cai X, Padoa-Schioppa C, Pasternak T, Seo H, Lee D, Wang X-J (2014) A hierarchy of intrinsic timescales across primate cortex. Nat Neurosci 17:1661–1663.

Nayebi A, Kong NCL, Zhuang C, Gardner JL, Norcia AM, Yamins DLK (2023) Mouse visual cortex as a limited resource system that self-learns an ecologically-general representation. PLOS Comput Biol 19:e1011506.

Niell CM (2011) Exploring the Next Frontier of Mouse Vision. Neuron 72:889–892.

Oude Lohuis MN, Marchesi P, Olcese U, Pennartz CMA (2024) Triple dissociation of visual, auditory and motor processing in mouse primary visual cortex. Nat Neurosci:1–14.

Piasini E, Soltuzu L, Muratore P, Caramellino R, Vinken K, Op de Beeck H, Balasubramanian V, Zoccolan D (2021) Temporal stability of stimulus representation increases along rodent visual cortical hierarchies. Nat Commun 12:4448.

Tafazoli S, Safaai H, De Franceschi G, Rosselli FB, Vanzella W, Riggi M, Buffolo F, Panzeri S, Zoccolan D (2017) Emergence of transformation-tolerant representations of visual objects in rat lateral extrastriate cortex. eLife 6:e22794.

References

Ballesteros-Yáñez, Inmaculada et al. (2006). “Density and morphology of dendritic spines in mouse neocortex.” *Neuroscience* 138.2, pp. 403–409.

Benavides-Piccione, Ruth et al. (2006). “Dendritic size of pyramidal neurons differs among mouse cortical regions.” *Cerebral Cortex* 16.7, pp. 990–1001.

Cadena, Santiago A et al. (2019). “How well do deep neural networks trained on object recognition characterize the mouse visual system?” In: *Real Neurons {\&} Hidden Units: Future directions at the intersection of neuroscience and artificial intelligence@ NeurIPS 2019.*

Chaudhuri, Rishidev et al. (Oct. 2015). “A Large-Scale Circuit Mechanism for Hierarchical Dynamical Processing in the Primate Cortex.” *Neuron* 88.2, pp. 419–431. ISSN: 1097-4199. DOI: 10.1016/j.neuron.2015.09.008.

D’Souza, Rinaldo David et al. (2016). “Recruitment of inhibition and excitation across mouse visual cortex depends on the hierarchy of interconnecting areas.” *Elife* 5, e19332.

DiCarlo, James J and David D Cox (2007). “Untangling invariant object recognition.” *Trends in cognitive sciences* 11.8, pp. 333–341.

DiCarlo, James J, Davide Zoccolan, and Nicole C Rust (2012). “How does the brain solve visual object recognition?” *Neuron* 73.3, pp. 415–434.

Elston, Guy N and Ichiro Fujita (2014). “Pyramidal cell development: postnatal spinogenesis, dendritic growth, axon growth, and electrophysiology.” *Frontiers in Neuroanatomy* 8, p. 78.

Fairhall, Adrienne L et al. (2001). “Efficiency and ambiguity in an adaptive neural code.” *Nature* 412.6849, pp. 787–792.

Fulcher, Ben D et al. (2019). “Multimodal gradients across mouse cortex.” *Proceedings of the National Academy of Sciences* 116.10, pp. 4689–4695.

Gilman, Joshua P, Maria Medalla, and Jennifer I Luebke (2017). “Area-specific features of pyramidal neurons—a comparative study in mouse and rhesus monkey.” *Cerebral Cortex* 27.3, pp. 2078–2094.

Glickfeld, Lindsey L and Shawn R Olsen (2017). “Higher-order areas of the mouse visual cortex.” *Annual review of vision science* 3, pp. 251–273.

Glickfeld, Lindsey L, R Clay Reid, and Mark L Andermann (2014). “A mouse model of higher visual cortical function.” *Current opinion in neurobiology* 24, pp. 28–33.

Harris, Julie A et al. (2019). “Hierarchical organization of cortical and thalamic connectivity.” *Nature* 575.7781, pp. 195–202.

Hasson, Uri, Janice Chen, and Christopher J Honey (2015). “Hierarchical process memory: memory as an integral component of information processing.” *Trends in cognitive sciences* 19.6, pp. 304–313.

Hasson, Uri, Eunice Yang, et al. (Mar. 2008). “A Hierarchy of Temporal Receptive Windows in Human Cortex.” *Journal of Neuroscience* 28.10, pp. 2539–2550. ISSN: 0270-6474, 1529-2401. DOI: 10.1523/JNEUROSCI.5487-07.2008. Available from: https://www.jneurosci.org/content/28/10/2539 (visited on 07/21/2020).

Heuvel, Jorge de et al. (Oct. 2020). “Characterizing spreading dynamics of subsampled systems with nonstationary external input.” *Phys. Rev. E* 102 (4), p. 040301. DOI: 10.1103/PhysRevE.102.040301. Available from: https://link.aps.org/doi/10.1103/PhysRevE.102.040301.

Hsu, Alexander, Jennifer I Luebke, and Maria Medalla (2017). “Comparative ultrastructural features of excitatory synapses in the visual and frontal cortices of the adult mouse and monkey.” *Journal of Comparative Neurology* 525.9, pp. 2175–2191.

Huntenburg, Julia M et al. (2021). “Gradients of functional connectivity in the mouse cortex reflect neocortical evolution.” *NeuroImage* 225, p. 117528.

Luebke, Jennifer I (2017). “Pyramidal neurons are not generalizable building blocks of cortical networks.” *Frontiers in neuroanatomy* 11, p. 11.

Lundstrom, Brian N et al. (2008). “Fractional differentiation by neocortical pyramidal neurons.” *Nature neuroscience* 11.11, pp. 1335–1342.

Mao, Tianyi et al. (2011). “Long-range neuronal circuits underlying the interaction between sensory and motor cortex.” *neuron* 72.1, pp. 111–123.

Matteucci, Giulio, Eugenio Piasini, and Davide Zoccolan (2024). “Unsupervised learning of mid-level visual representations.” *Current opinion in neurobiology* 84, p. 102834.
Matteucci, Giulio and Davide Zoccolan (2020). “Unsupervised experience with temporal continuity of the visual environment is causally involved in the development of V1 complex cells.” *Science Advances* 6.22, eaba3742.
Młynarski, Wiktor F and Ann M Hermundstad (2021). “Efficient and adaptive sensory codes.” *Nature Neuroscience* 24.7, pp. 998–1009.
Morcos, Ari S and Christopher D Harvey (2016). “History-dependent variability in population dynamics during evidence accumulation in cortex.” *Nature neuroscience* 19.12, pp. 1672–1681.
Muratore, Paolo et al. (2022). “Prune and distill: similar reformatting of image information along rat visual cortex and deep neural networks.” *Advances in Neural Information Processing Systems* 35, pp. 30206–30218.
Murray, John D. et al. (Dec. 2014). “A Hierarchy of Intrinsic Timescales across Primate Cortex.” *Nature Neuroscience* 17.12, pp. 1661–1663. ISSN: 1546-1726. DOI: 10.1038/nn.3862. Available from: https://www.nature.com/articles/nn.3862 (visited on 07/03/2019).
Nayebi, Aran et al. (2023). “Mouse visual cortex as a limited resource system that self-learns an ecologically-general representation.” *PLOS Computational Biology* 19.10, e1011506.
Niell, Cristopher M (2011). “Exploring the next frontier of mouse vision.” *Neuron* 72.6, pp. 889–892.
Panzeri, Stefano et al. (2022). “The structures and functions of correlations in neural population codes.” *Nature Reviews Neuroscience* 23.9, pp. 551–567.
Piasini, Eugenio et al. (2021). “Temporal stability of stimulus representation increases along rodent visual cortical hierarchies.” *Nature communications* 12.1, p. 4448.
Pinto, Lucas, David W Tank, and Carlos D Brody (2022). “Multiple timescales of sensory-evidence accumulation across the dorsal cortex.” *Elife* 11, e70263.
Rao, Rajesh PN and Dana H Ballard (1999). “Predictive coding in the visual cortex: a functional interpretation of some extra-classical receptive-field effects.” *Nature neuroscience* 2.1, pp. 79–87.
Riesenhuber, Maximilian and Tomaso Poggio (1999). “Hierarchical models of object recognition in cortex.” *Nature neuroscience* 2.11, pp. 1019–1025.
Rossi-Pool, Román et al. (2021). “Invariant timescale hierarchy across the cortical somatosensory network.” *Proceedings of the National Academy of Sciences* 118.3, e2021843118.
Runyan, Caroline A et al. (2017). “Distinct timescales of population coding across cortex.” *Nature* 548.7665, pp. 92–96.
Siegle, Joshua H et al. (2021). “Survey of spiking in the mouse visual system reveals functional hierarchy.” *Nature* 592.7852, pp. 86–92. DOI: 10.1038/s41586-020-03171-x.
Simoncelli, Eero P (2003). “Vision and the statistics of the visual environment.” *Current opinion in neurobiology* 13.2, pp. 144–149.
Tafazoli, Sina et al. (2017). “Emergence of transformation-tolerant representations of visual objects in rat lateral extrastriate cortex.” *Elife* 6, e22794.
Valente, Martina et al. (2021). “Correlations enhance the behavioral readout of neural population activity in association cortex.” *Nature neuroscience* 24.7, pp. 975–986.
Wang, Quanxin, Enquan Gao, and Andreas Burkhalter (2011). “Gateways of ventral and dorsal streams in mouse visual cortex.” *Journal of Neuroscience* 31.5, pp. 1905–1918.
Wang, Quanxin, Olaf Sporns, and Andreas Burkhalter (2012). “Network analysis of corticocortical connections reveals ventral and dorsal processing streams in mouse visual cortex.” *Journal of Neuroscience* 32.13, pp. 4386–4399.
Wilting, J. and V. Priesemann (June 2019). “Between Perfectly Critical and Fully Irregular: A Reverberating Model Captures and Predicts Cortical Spike Propagation.” *Cerebral Cortex* 29.6, pp. 2759–2770. ISSN: 1047-3211. DOI: 10.1093/cercor/bhz049. Available from: https://academic.oup.com/cercor/article/29/6/2759/5476016 (visited on 08/05/2020).
Wilting, Jens, Jonas Dehning, et al. (2018). “Operating in a Reverberating Regime Enables Rapid Tuning of Network States to Task Requirements.” *Frontiers in Systems Neuroscience* 12. ISSN: 1662-5137. DOI:

10.3389/fnsys.2018.00055. Available from: https://www.frontiersin.org/articles/10.3389/fnsys.2018.00055/full (visited on 07/21/2020).

Wilting, Jens and Viola Priesemann (June 2018). "Inferring Collective Dynamical States from Widely Unobserved Systems." *Nature Communications* 9.1, p. 2325. ISSN: 2041-1723. DOI: 10.1038/s41467-018-04725-4. Available from: https://www.nature.com/articles/s41467-018-04725-4 (visited on 08/05/2020).

Zeraati, Roxana et al. (2023). "Intrinsic timescales in the visual cortex change with selective attention and reflect spatial connectivity." *Nature communications* 14.1, p. 1858.
